# Spatiotemporal variation in the microbiome of *Aedes vexans* from Korea reveals regional markers linked to environmental risk factors

**DOI:** 10.1128/spectrum.02587-25

**Published:** 2026-03-31

**Authors:** Abdulkadir Yusif Maigoro, Jeong Hyeon Lee, Da-Reum Heo, Bo-Ram Yun, Hee Il Lee, Hyung-Wook Kwon

**Affiliations:** 1 Convergence Research Center for Insect Vectors, Yeonsu-gu, Incheon, Republic of Korea; 2 Department of Life Sciences, Incheon National University 34958, Yeonsu-gu, Incheon, Republic of Korea; 3 Division of Vectors and Parasitic Diseases, Korea Disease Control and Prevention Agency 204189, Chunbuk, Cheongju, Republic of Korea; National Chung Hsing University, Taichung, Taiwan

**Keywords:** microbiome, *Aedes vexans*, West Nile virus, *Wolbachia*, *Dickeya*

## Abstract

*Aedes vexans* is a widespread mosquito species known to carry West Nile virus (WNV); however, our understanding of how its microbiome changes across different regions and seasons, particularly in temperate areas such as South Korea, remains limited. In this study, we examined the microbiome of *Aedes vexans* collected from 16 locations over 3 consecutive summer months. Using 16S rRNA sequencing, we found that the microbiome was largely made up of Proteobacteria, but the specific genera present, like *Dickeya, Spiroplasma,* and members of *Enterobacterales,* varied depending on the location and time of collection. *Dickeya*, in particular, was more common in inland areas and stayed relatively stable over time, which suggests it could serve as a useful microbial marker. We also observed a significant absence of *Wolbachia*, a common endosymbiont in mosquitoes, which hypothesizes potential increased risk of WNV transmission. Diversity analyses showed clear differences in microbial communities by region, and we found seasonal patterns in genera like *Asaia* and *Pseudomonas*, which were correlated to mosquito abundance and local environmental conditions. These patterns held up when we looked at co-occurrence networks between microbes. Altogether, this is the first study to track *Aedes vexans* microbiome across both space and time in Korea, and our findings offer new insights into mosquito ecology and the potential use of bacteria in disease control strategies.

**IMPORTANCE:**

Understanding the dynamics of the mosquito microbiome is essential for predicting disease risk and developing targeted vector control strategies. *Aedes vexans*, a globally distributed species and potential vector for West Nile virus (WNV), has seen a notable population increase in South Korea, yet its microbial ecology remains poorly characterized. This study provides the first comprehensive spatiotemporal analysis of *Aedes vexans* microbiota across Korea, identifying key microbial taxa that vary by region and season. The absence of *Wolbachia*, a known antiviral symbiont, and the dominance of *Dickeya,* a plant-associated genus with potential ecological implications, underscore the need for microbiome-informed surveillance tools. By highlighting native microbial signatures and their environmental drivers, this work lays the groundwork for microbiota-based monitoring of vector populations and opens new avenues for symbiont-based interventions in arbovirus control.

## INTRODUCTION

West Nile virus (WNV) is an arthropod-borne virus (arbovirus) that poses a significant public health concern, causing disease in humans and other vertebrates through mosquito-mediated transmission (1). First isolated in 1999, WNV has since spread globally, with its primary transmission cycle involving mosquito vectors and avian reservoirs (2). While *Culex* species are considered the primary vectors of WNV, *Aedes vexans* has emerged as a secondary yet important vector, particularly due to its ecological abundance and aggressive biting behavior toward humans, wild, and domestic animals (3, 4). This species exhibits moderate susceptibility to WNV, with some individuals developing disseminated infections capable of efficient virus transmission (5). Notably, humans face a higher risk of being bitten by infected *Aedes vexans* compared to *Culex* species, with infection rates reaching up to 73.6% (6).

In recent years, the population of *Aedes vexans* in Korea has shown a dramatic increase of 3.0- to 7.8-fold since 2011 (7). This can indirectly be related to the species’ viral identifications. This trend may be associated with the potential for arboviral transmission, exemplified by the identification of *Panmunjeom flavivirus* in *Aedes vexans nipponii* near Korea’s Demilitarized Zone (8). Therefore, the growing evidence of *Aedes vexans* may signify an increasing risk for the transmission of virus-mediated diseases in both humans and animals. Although suspected to be the second most prevalent mosquito species in Korea, its official ranking is not fully elucidated (9).

Mosquito-borne pathogen transmission is shaped by complex interactions among the mosquito vector, the pathogen, and the vertebrate host. Following a blood meal or nectar feeding, the pathogen enters the mosquito’s midgut, replicates, and disseminates to secondary tissues such as the fat body and nerve tissues, ultimately reaching the salivary glands for subsequent bites (10). At the core of this transmission dynamic lies the mosquito’s midgut microbiota, which plays a crucial role in modulating vector competence (11).

This chain of interactions between the vector and host, along with other factors like the increased emergence of the vector (*Aedes vexans*) in various regions, especially water bodies, seasonality, and variability in agricultural landscapes, underscores the necessity to study this species within Korea. It is important to emphasize that mosquito-borne diseases in the Republic of Korea have a unique epidemiology due to the rapid improvements in hygiene and economic status, the occurrence of four distinct seasons, and separation from North Korea (12).

Recent evidence suggests that the mosquito microbiome significantly influences the susceptibility to infection by regulating immune responses and interacting directly with invading pathogens (11). Microbial communities colonize various mosquito tissues, including the gut, salivary glands, and reproductive organs, and these communities vary by species, even under uniform rearing conditions, highlighting species-specific microbial profiles (12, 13). In *Aedes* mosquitoes, microbiome establishment begins early from parents to offspring via eggs (14). Among the vertically transmitted microbes, *Wolbachia*, a widely studied endosymbiont, has gained attention for its ability to modulate pathogen replication and transmission in mosquitoes (15). Some other species naturally have *Wolbachia* as a dominant symbiont, whereby differences in microbial community composition between the species may be correlated with the capacity to transmit pathogens like WNV (16). Other studies show that gut microbiota can modulate vector competence via immune modulation, gut barrier, direct competition, among others. Supporting the idea that *Wolbachia* being part of the microbiome plays a role in risk (17).

Depending on the host-pathogen system, *Wolbachia* can either enhance or inhibit viral replication. For example, it suppresses dengue virus in *Aedes aegypti* and *Aedes albopictus,* yet enhances WNV replication in *Aedes aegypti* cell lines while interfering with virus assembly (18–20). Due to its antiviral properties, *Wolbachia* has been exploited in vector control programs by releasing artificially infected mosquitoes into endemic regions to reduce arbovirus transmission (e.g., eliminatedengue.com) (21).

Given this background, environmental and ecological factors are crucial determinants of the mosquito microbiome composition. During the larval stage, water chemistry, population density, and food availability shape early microbial communities (22). In adulthood, both intrinsic and extrinsic factors, including geography, diet, and climate, influence microbial diversity. Global studies have shown significant variation in mosquito microbiota at lower taxonomic levels (genus and species), even when phylum-level profiles remain relatively consistent (23). These regional microbial signatures may partly explain global differences in arboviral disease patterns (2). Similar to other studies, different locations contribute to shaping the mosquito’s microbiota, underscoring the importance of sample location (1). In an interesting finding, Fausta et al. compared the microbiota of *Aedes spp.* from various countries with an emphasis on identifying unique bacteria and provided new insights into the spatial variation of the bacterial communities in mosquitoes (24). Apart from locations, a type of vector-host interaction also contributed to identifying particular taxa. For example, bacterial communities associated with blood-fed and starved bed bugs show a dominance of *Wolbachia* and *Dickeya* at the genus level, respectively (25). These genera act as animal and plant pathogens, respectively.

In this study, we investigated the microbiome of *Aedes vexans* mosquitoes collected from 16 ecologically distinct locations across South Korea. Using 16S rRNA gene-based microbiome analysis, we examine how geographic location, environmental variables (e.g., temperature and humidity), and seasonal variation shape microbial diversity and composition. Special attention is given to the presence of potentially pathogenic taxa and their implications for WNV transmission, as well as the influence of regional and seasonal shifts, a dimension yet to be fully characterized in *Aedes vexans* populations in Korea.

## MATERIALS AND METHODS

### Sample collection and identification

**Fig 1:**
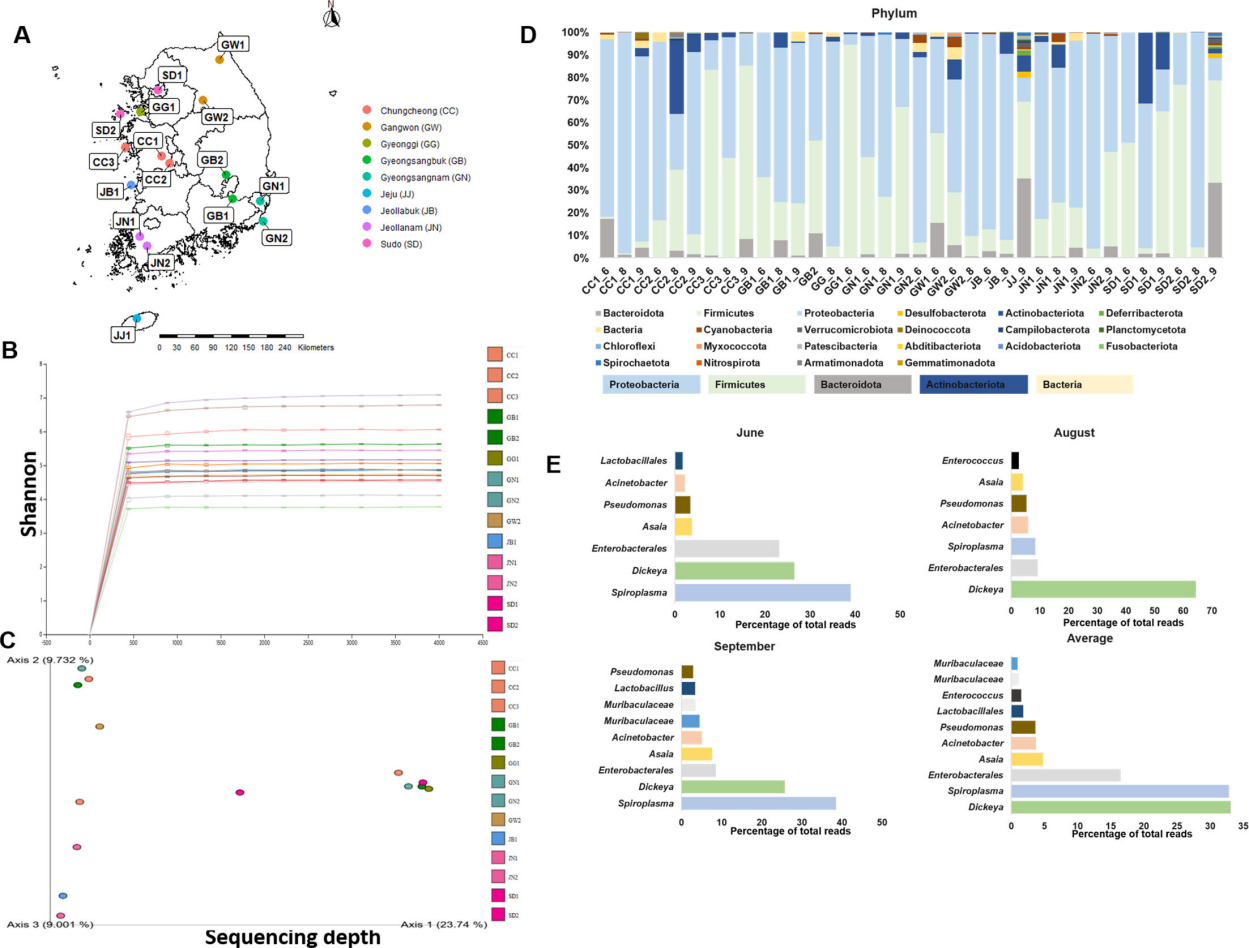
(**A**) Sites sampled for *Aedes vexans* in South Korea. Sixteen (16) different mosquito collection sites were selected based on the incidence of reported malaria cases and the area of water bodies prone to West Nile viral (WNV) infection. Multiple sampling sites were utilized in certain regions with high mosquito collections to identify any potential pathogen effect through microbiome-environment interaction. The full meaning of each region is as follows: Gangwon 1 (GW1), Gangwon 2 (GW2), Gyeonggi (GG), Gyeongnam 1 (GN1), Gyeongnam 2 (GN2), Gyeongbuk 1 (GB1), Gyeongbuk 2 (GB2), Sudo 1 (SD1), Sudo 2 (SD2), Jeonnam 1 (JN1), Jeonnam 2 (JN2), Jeonbuk (JB), Jeju (JJ), Chungcheong 1 (CC1), Chungcheong 2 (CC2), and Chungcheong 3 (CC3). (**B**) Rarefaction graph showing the number of reads after denoising. (**C**) Score plot for principal coordinate analysis (PCoA) of the bacterial community compositions at the genus level in the *Aedes vexans* using a multivariate analysis method. The individual samples from each month had different shapes and were color-coordinated to the respective region. A color gradient used ranges from the Brown start, for the CC1 region, to the Purple cube, for SD2. (**D**) Overall microbiome composition by sampling sites at the phylum, order, and genus levels. At the phylum level, Proteobacteria, Firmicutes, and Bacteroidota are the dominant taxa. (**E**) Average microbiome distribution based on months of sampling. These include June, August, and September. A total average of the most abundant genera was identified based on the sampling months and presented as an average. The bar size represents the relative abundance of each taxon. Analysis was performed using QIIME2 and R-software.

Adult *Aedes vexans* mosquitoes were collected using Biogent Sentinel traps and Black light traps, which are effective for capturing both host-seeking and nocturnally active mosquitoes. The collections were conducted collaboratively by the Vector Analysis Division of the Korea Disease Control and Prevention Agency (KDCA) and 16 regional vector surveillance centers distributed across South Korea (Fig. 1A). Species identification was performed visually using standard morphological keys, and individuals were categorized by sex and developmental stage (26). Only adult mosquitoes collected from ecologically comparable environments were included in the analysis to ensure consistency. Mosquitoes were sampled from 16 sites across 8 provinces and metropolitan areas in South Korea, including Chungcheong-do (CC), Gangwon-do (GW), Gyeonggi-do (GG), Gyeongsangbuk-do (GB), Gyeongsangnam-do (GN), Jeju-do (JJ), Jeollabuk-do (JB), Jeollanam-do (JN), and the greater Sudogwon area (SD). Sampling was conducted during three distinct seasonal periods: June (early summer), August (peak summer), and September (early autumn) to capture temporal variations in environmental conditions, such as temperature and precipitation. In addition, the September data set was used to assess qualitative shifts in microbial community composition. Metadata from each collection was categorized by month for downstream comparative analysis (Tables S1 to S4).

### DNA extraction

Genomic DNA was extracted from the whole bodies of adult *Aedes vexans* mosquitoes using the QIAGEN PowerSoil Kit (47014; QIAGEN, Hilden, Germany), following the manufacturer’s protocol. This will enable us to capture a more comprehensive microbial community profile while still enabling comparisons across groups (27). Before extraction, all mosquito specimens were stored at –80°C to preserve DNA integrity. To minimize the risk of contamination, all workspaces and equipment used during the extraction process were sterilized with 80% ethanol. DNA concentrations were measured for each region and collection period using a NanoDrop 2000 UV spectrophotometer (Thermo Scientific, Branchburg, NJ, USA). DNA quality was further confirmed via 1% agarose gel electrophoresis (22). Detailed concentration values for all samples are provided in Table S5A through D.

### 16S sequencing and taxonomic analysis

For all samples, primers were designed to amplify the V3-V4 region of the 16S ribosomal DNA. The 16S rRNA V3-V4 region was amplified using PCR and confirmed by electrophoresis. The following were the PCR conditions: 3 min at 96°C, then 30 cycles of 30 s at 96°C, 30 s at 55°C, 30 s at 72°C, and finally 5 min at 72°C. The 16S rRNA V3-V4 region was amplified, and the appearance of a ladder between 300 and 400 bp (base pair) using 1.2% agarose gels indicated successful amplification (Table S5D). Ampure XP beads (Beckman, USA) were used to purify amplified DNA from each sample. According to the content of DNA and molecular weight, samples were pooled in identical quantities and utilized to create Illumina DNA libraries. The libraries were then sequenced in Illumina MiSeq runs to obtain 2 × 300 bp paired-end reads. Raw data generated through sequencing were used to analyze microbial communities using QIIME2 v.2024.2 (23). The denoised-paired command was used to rectify errors, eliminate chimeras, and integrate paired-end reads using the DADA2 plugin in QIIME2 (23, 28). Chimeric sequences that could not be definitively linked to an operational taxonomic unit (OTU) were eliminated. OTU is a scientific way of grouping DNA sequences from microbial communities based on their similarities. In this article, a USEARCH algorithm was used, considering its efficiency in implementation. OTUs that could not be taxonomically categorized were labeled “unclassified” and were excluded from further analysis. Based on the diversity data, taxonomy classification and differential abundance analysis were used to classify microorganisms from the phylum to the genus level and interpret differences between microorganisms to analyze the characteristics and correlations of microbial communities by region and species. For comparison, the ratio data were subdivided into different locations and months. On the other hand, data comparison was performed using secondary data obtained. The raw sequence data are available at the European Bioinformatics Institute database under accession number ERP021438. The data set is also available at https://qiita.ucsd.edu/ (ID 10815).

### Bacterial profiles

From the filtered data and at the phylum to genus levels, read count and abundance data for the bacterial OTUs were evaluated. Species-level classification was not performed due to the limitation of resolution in 16S-based analyses. Low-abundant taxa with less than 1% relative abundance values were grouped into the “<1%” category. R packages such as ggplots and Pheatmap were used for heatmap construction. The NetworkD3 R package was used for Sankey diagram construction.

### Statistical analysis

The Shannon diversity, Jaccard, and the Bray–Curtis dissimilarity index were used to analyze microbial diversity within (alpha diversity) and between (beta diversity) samples in QIIME2. The average Shannon indices were reported and compared between samples using pairwise Kruskal–Wallis tests with Benjamini–Hochberg FDR corrections for multiple comparisons. The results of the Jaccard and Bray–Curtis dissimilarity indices were compared between samples using paired PERMANOVA tests (999 permutations) with FDR corrections. The statistical analyses described above were performed using R version 4.1.0 in RStudio Version 1.4.1106, and QIIME2 (29) as adopted from Lee et al. (24). A correlation matrix was performed for Microbial Co-occurrence Network using Bayesian Model in RStudio version R.4.4.2 using various packages such as igraph, Hmisc, reshape2, and ggplot2. Both r = 0.5 and *P*-value < 0.05 were used as thresholds for significant values.

## RESULTS

### Microbiome profiles of *Aedes vexans* from different regions in Korea

A total of 1,861 adult *Aedes vexans* mosquitoes were collected from 16 regions across Korea. A higher number of mosquitoes was collected in June (1,088), followed by August (599) and lastly September (174). A total of 36 samples were used for microbiome analysis, with up to a maximum of 10 mosquitoes used per sample. The mosquito microbiome was analyzed by region and collection date (Fig. 1A).

Next-generation sequencing (NGS) returned an average of 72.96%–83.67% of reads (Avg [Q ≥ 30]). Overall, a total of 1,988,731, 1,031,419, and 913,364 reads were obtained for June, August, and September samples, respectively Tables S6 to S8. On average, 68% of valid reads were filtered out. Rarefaction graph provided evidence that sufficient sampling effort was carried out, with each sample represented by a distinct color (Fig. 1B). The resulting rarefaction curve provided evidence that the 16 sample reads have shown a satisfactory depth. Results revealed that bacterial alpha diversity was significantly higher among samples from the GW2 region, followed by samples from the JJ region, with samples from the GG region as the least diverse (Table S9).

Furthermore, when examining the PCoA graph, the observed clustering patterns were statistically significant (*P* < 0.05). Despite the change in the collection date, certain regions clustered together, such as CC2, CC3, GN1, and GW2. Other regions changed with the change in sampling months, including GB1 and SD2. On the other hand, JN, JN2, SD1, CC, and JB were the identified regions that clustered at least once during the collection dates (Fig. 1C).

Data at the phylum level were analyzed to identify various OTUs present in all mosquitoes and in the 16 regions during the selected months. Overall, Proteobacteria appeared to be the most abundant and widely distributed phylum, followed by Firmicutes, Bacteroidota, and Actinobacteria (Fig. 1D). On the other hand, samples in axis 2 × 3 had a higher proportion of Actinobacteria and Bacteroidota as compared to other samples. Axes 2 and 3 had similar microbiome compositions, although axis 2 samples contained small amounts of Cyanobacteria, with a slightly higher proportion of Firmicutes. For axis 1 of the PCoA of beta diversity, their samples have an average higher proportion of Firmicutes than Proteobacteria. After critical observations, we found that samples with a higher proportion of Proteobacteria (GN1_8, JN1_6, and JN2_9), among others, are found located closer to axes 2 and 3 (Fig. S1).

### Spatial and seasonal variation of microbiome

There was variation in the microbiome distribution based on region of sampling as well as based on time (season) of sampling. In this work, the time of sampling was categorized into three, *viz* June (6), August (8), and September (9). In June and August, *Spiroplasma*, *Dickeya,* and *Enterobacterales* were the most abundant taxa based on average distribution, respectively. On the other hand, *Dickeya* and *Enterobacterales* were the most abundant taxa in August, with an average abundance value of 43.64 and 7.79, respectively. Overall average abundance rate of the most abundant taxa was calculated, presenting *Dickeya* as the most abundant with an average value of 27.03, followed by *Spiroplasma* 19.28, and Enterobacterales with an average value of 13.16 (Fig. 1E).

**Fig 2:**
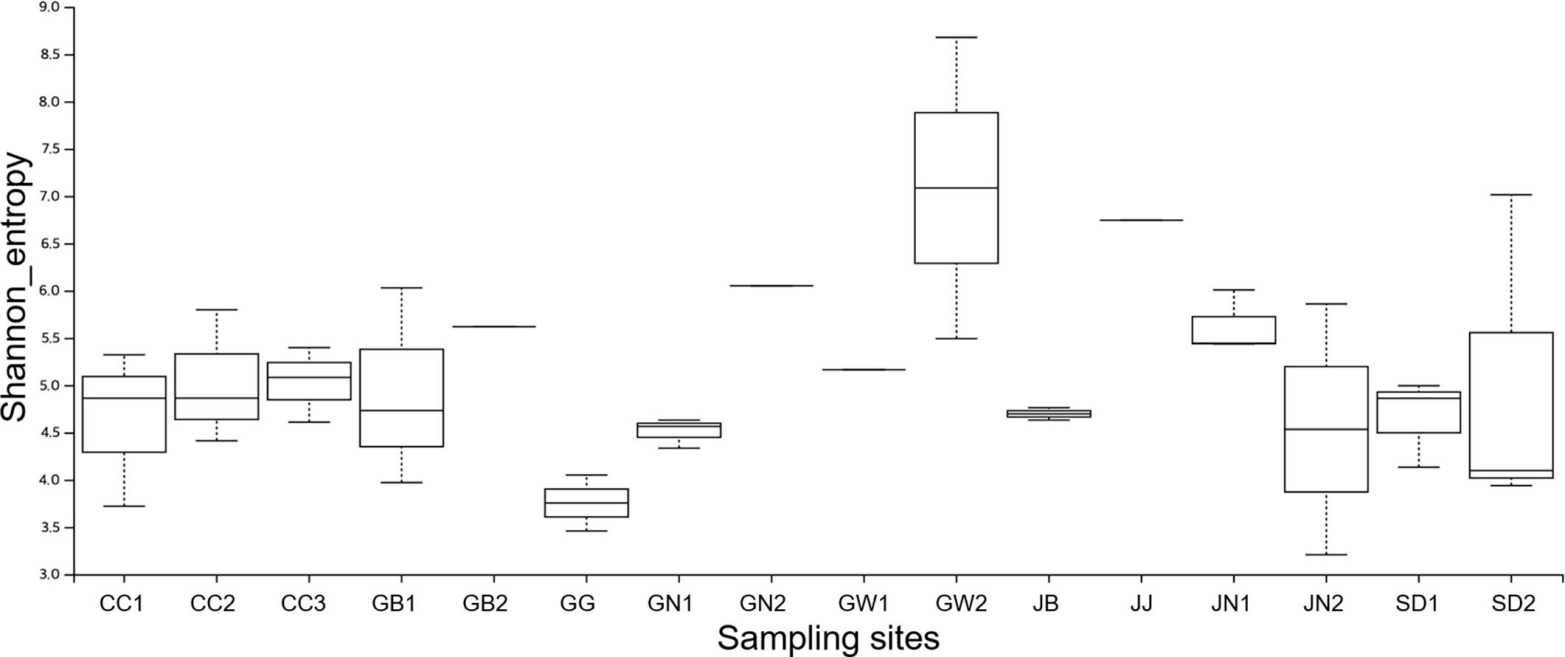
The Shannon entropy diversity index was calculated using statistical analysis to measure the degree of randomness of the microbiome diversity within each sample based on the species diversity and species richness of each sample. The box plot indicates the diversity between the samples of each region with one another using integrated packages built in the QIIME2 software.

Furthermore, metrics of alpha and beta diversity were subsequently assessed. Alpha diversity was used to analyze the microbial communities and their relationships. It revealed substantial differences in bacterial community diversity across mosquito groups (Fig. 2). GW2 exhibited the highest diversity, with a median Shannon index of approximately 7.0, suggesting a rich and evenly distributed bacterial community. On the other hand, GW10 and GW12 showed the lowest diversity (median ~4.0), with GW10 displaying very little variation among samples, while GW12 showed broader variability, indicating heterogeneity within the group. Other sampling groups, such as GW3, GW4, and GW7, show moderate richness and evenness of microbial communities, with the median value of (4.5–5.5). On the other hand, GW8 and GW9 had comparatively stable but lower diversity, whereas GW6 displayed both high values and considerable variation among other replicates. Overall, these findings demonstrate that mosquito microbiome diversity is group-dependent, with GW2 standing out as the most diverse community and GW10 and GW12 as the least diverse (Fig. 2).

### Microbiome variation by sampling site and collection period

Microbiome analysis revealed pronounced geographic and seasonal variation across sampling sites. From our data, the most prevalent bacterial groups were *Dickeya, Spiroplasma,* and *Enterobacterales,* with their relative abundances varying by region and season (Fig. S3). For example, *Dickeya* dominated in GW2 and JN1, while *Spiroplasma* was abundant in GB (June) and SD (June and August) but absent from several CC sites. On the other hand, *Asaia* was highly represented in GN2 and increased notably in CC1 and GB1. Site-specific differences were further reflected in JJ Island samples (September only), which showed distinct phylum-level profiles compared to mainland regions.

**Fig 3:**
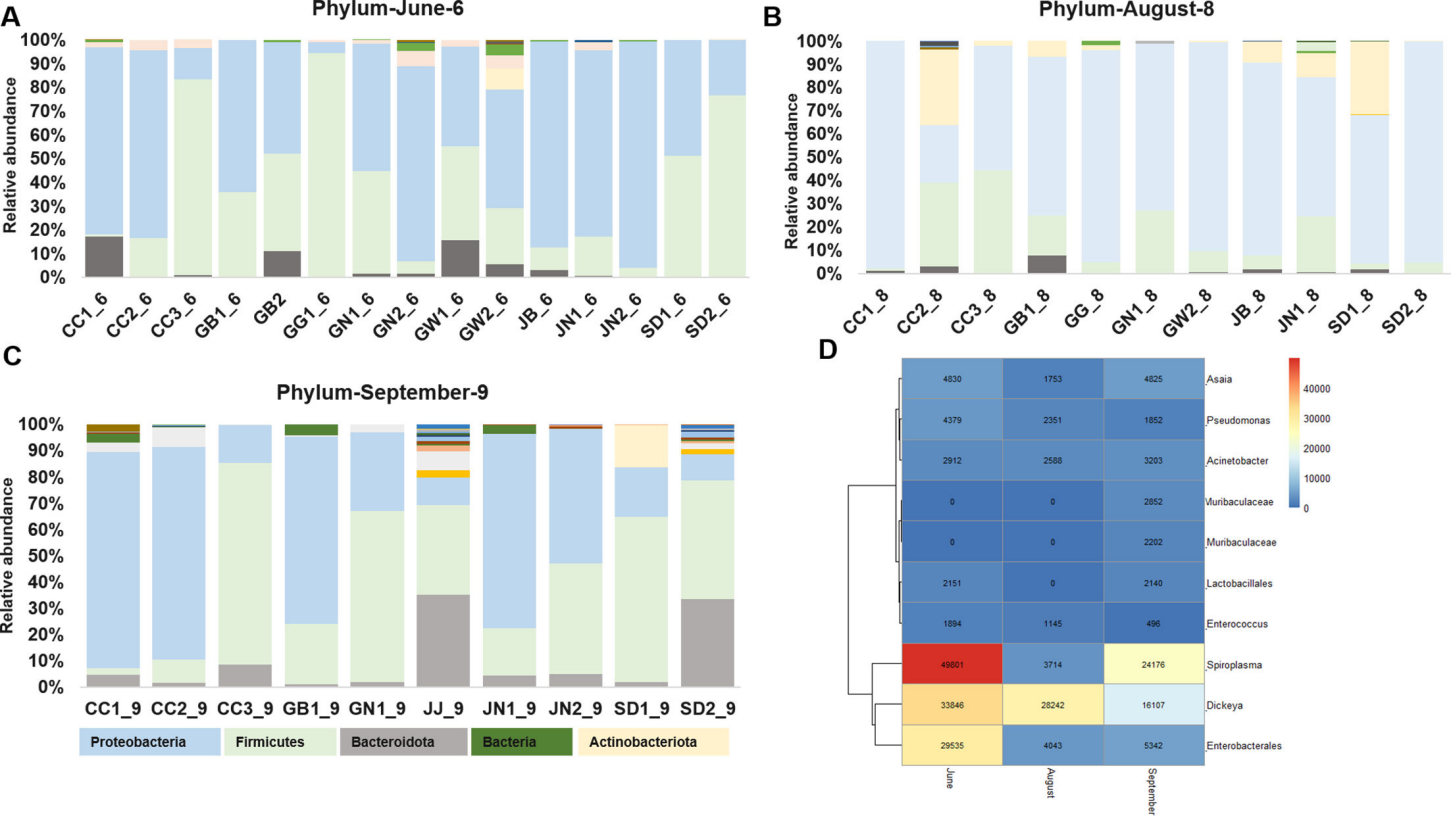
(**A–C**) Overall microbiome composition by sampling period at the phylum level. In June, Proteobacteria, Firmicutes, and Bacteroidota are the dominant taxa. In August, in addition to Actinobacteriota, Proteobacteria, Firmicutes, and Bacteroidota are the dominant taxa. While in September, Proteobacteria, Firmicutes, and Bacteroidota are the dominant taxa, which is similar to the June samples. JJ samples show a unique microbial distribution. The main regions include the following: Gangwon (GW), Gyeonggi (GG), Sudogwon (SW), Chungcheong (CC), Gyeongbuk (GB), Jeonbuk (JB), Gyeongnam (GN), Jeonnam (JN), and Jeju (JJ). Jeju samples show an entirely different pattern of bacterial composition as compared with the other regions. Microbiome accounting for less than 1% was considered less prevalent, and such was summed up to the category of <1%. (**D**) Heatmap generated using the Pheatmap R-package, indicating the average genus distributions during three different sampling periods, including June, August, and September. *Spiroplasma* has the highest average distribution in the months of June and September.

Seasonal dynamics were evident across sites, as shown in (Fig. 3A through C). Proteobacteria and Firmicutes remained dominant throughout, but June samples displayed higher diversity with substantial contributions from Bacteroidota (e.g., CC1, GB2, GN2)**,** whereas August samples showed a significant reduction in diversity, with Proteobacteria strongly dominating. On the other hand, September samples exhibited the broadest diversity, including Bacteroidota and Actinobacteriota spread in several regions (e.g., JJ, JN, SD). A heatmap (Fig. 3D) further illustrated these dynamics, showing *Asaia, Acinetobacter,* and *Muribaculaceae* as broadly distributed across samples, while dot plot analysis highlighted *Spiroplasma* and *Dickeya* as the most abundant genera.

Overall, these patterns suggest temporal restructuring of bacterial communities potentially linked to seasonal or environmental drivers. Importantly, statistical comparisons using PERMANOVA (*P* < 0.05) confirmed significant differences in beta diversity by both region and season, supporting our observed compositional shifts.

**Fig 4:**
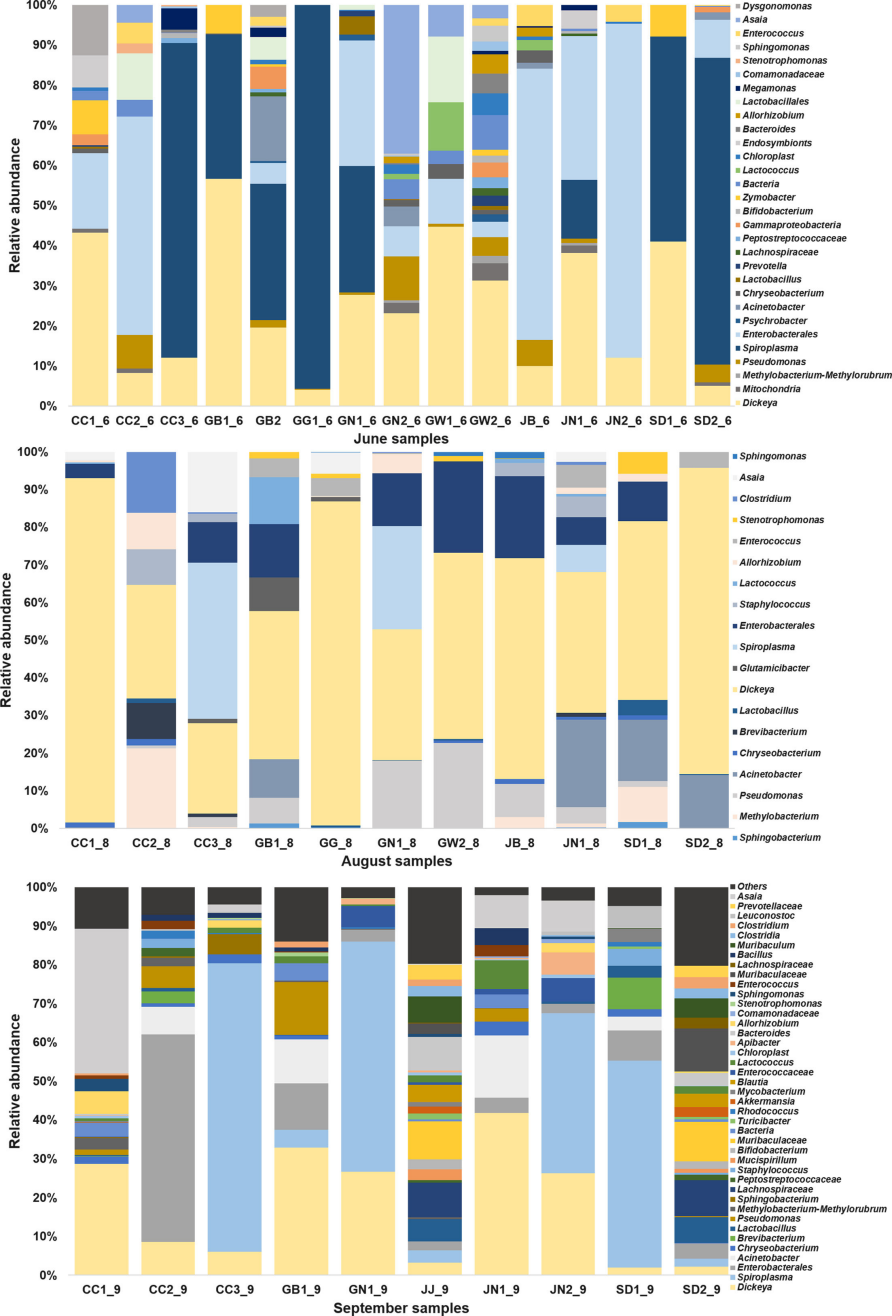
(June samples–September sample) Overall microbiome composition by sampling period at the genus level. (June samples) Representative samples were collected in June. At the genus level, dominant microbiome members were *Dickeya*, *Spiroplasma*, and *Enterobacterales*. (August samples) Representative samples were collected in August. At the genus level, dominant microbiome members were *Dickeya*, *Spiroplasma*, and *Enterobacterales,* which are relatively similar to the June samples. (September samples) Representative samples were collected in September. At the genus level, dominant microbiome members were *Dickeya*, *Spiroplasma*, and *Enterobacterales*. The main regions include the following: Gangwon (GW), Gyeonggi (GG), Sudogwon (SW), Chungcheong (CC), Gyeongbuk (GB), Jeonbuk (JB), Gyeongnam (GN), Jeonnam (JN), and Jeju (JJ). Jeju samples obtained in September show an entirely different pattern of bacterial composition as compared with the other regions. Microbiome accounting for less than 1% was considered less prevalent and thus summed up to the category of <1%.

At the genus level, bacterial communities showed clear temporal restructuring across June, August, and September, respectively (Fig. 4). June samples displayed moderate diversity, and they were dominated by *Lactobacillus* or *Enterococcus,* with frequent distribution of *Pseudomonas, Sphingomonas,* and *Asaia*. In August, diversity decreased, with the majority of the samples dominated by one or two genera (e.g., *Lactobacillus, Enterococcus, Clostridium*), suggesting a connection toward a more specialized community structure. However, in September, richness and evenness increased markedly, with additional genera such as *Prevotellaceae, Blautia,* and *Staphylococcus* emerging, in addition to a broader representation of low-abundance taxa. Collectively, these results indicate dynamic and seasonally dependent shifts in genus-level community composition, with early and mid-season dominated by fewer taxa, and late season characterized by higher diversity and ecological balance.

**Fig 5:**
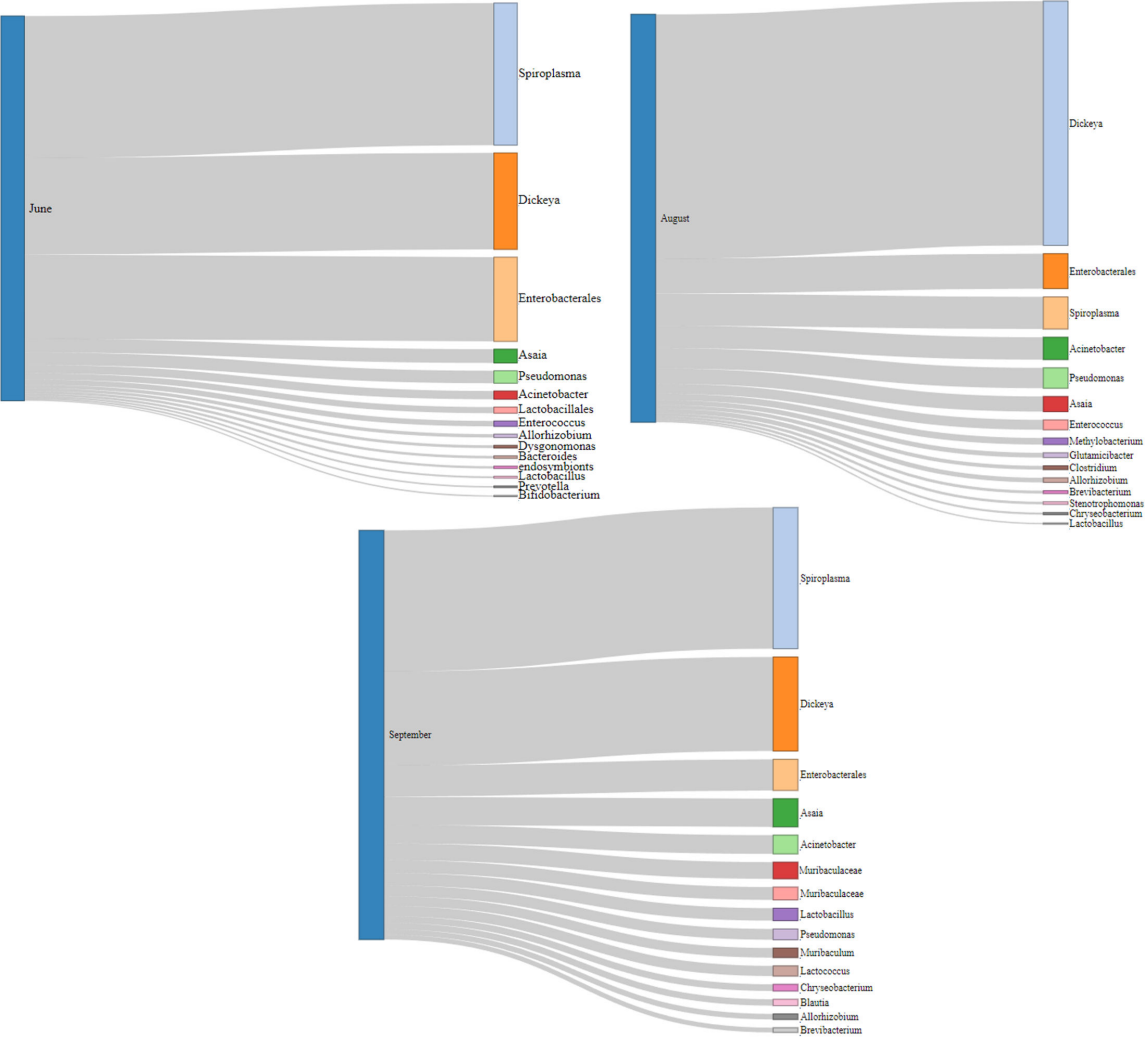
Sankey diagrams were generated using the NetworkD3 R package. (June) Sankey diagram showing an illustration of the correlation between a sampling period (June) and each bacterial genus. (August) Sankey diagram showing an illustration of the correlation between a sampling period (August) and each bacterial genus. (September) Sankey diagram showing an illustration of the correlation between a sampling period (September) and each bacterial genus. The lines connecting sampling periods are called nodes. The width of each node represents the taxa’s relative abundance.

Furthermore, when taxa were compared across seasons irrespective of sampling location, clear temporal shifts were observed, as shown in Fig. 5. June samples were dominated by *Spiroplasma, Dickeya,* and *Enterobacterales,* with less abundance from *Asaia* and *Pseudomonas*. August samples showed that *Dickeya* remained abundant with an increase in diversity and distribution of other taxa such as *Spiroplasma* and *Dickeya* alongside new contributors including *Muribaculaceae, Lachnospiraceae,* and *Blautia*.

### Regional-specific microbial distribution at the genus level

**Fig 6:**
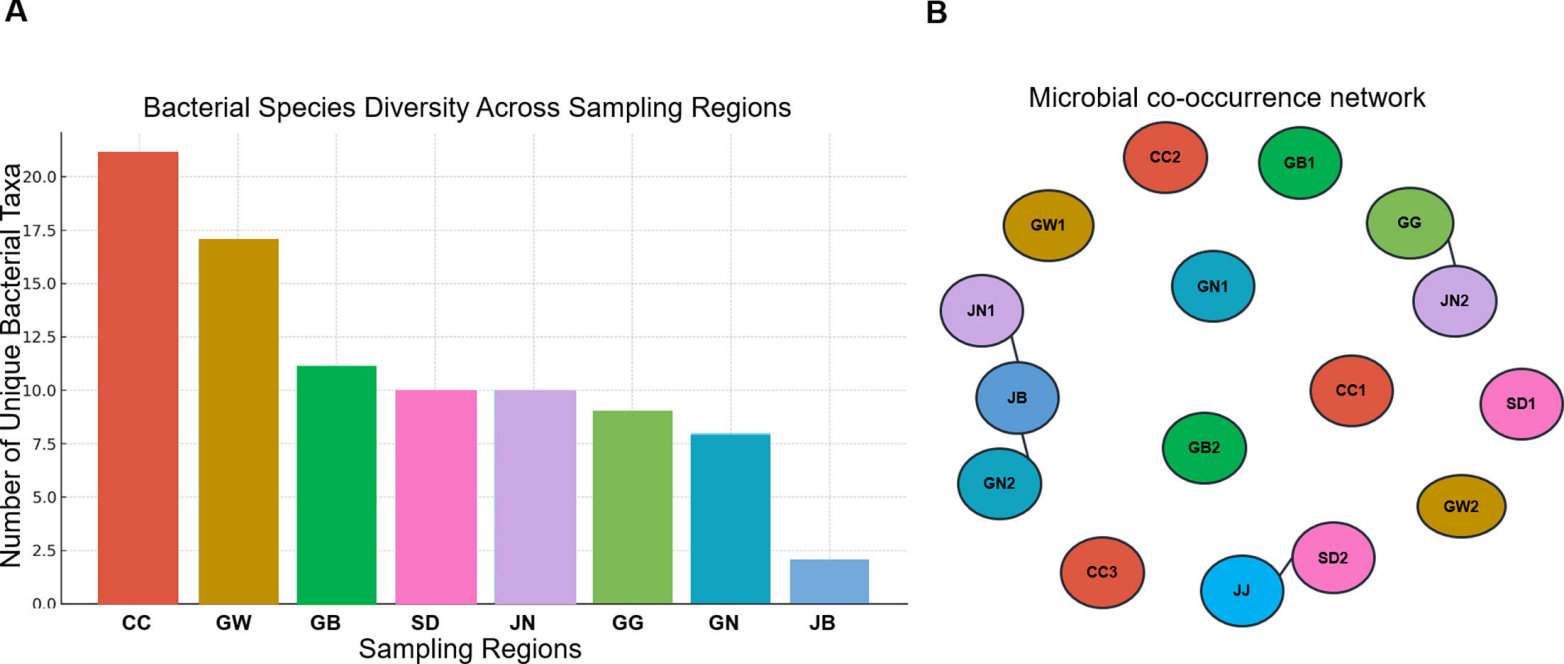
(**A**) Number of unique taxa distributed within various sampling sites. (**B**) A microbial co-occurrence network was generated using Bayesian network analysis in R-software, indicating a significant correlation between the microbiome at the genus level and various sampling locations. Sampling locations with similar environmental conditions tend to share similar microbial distributions. The nodes represent sampling locations, while the edges connecting the nodes represent correlations between the significant nodes.

At the genus level, specific microbiome biomarkers were identified based on eight different regions (Table S1). The CC region exhibited the highest taxa diversity, encompassing approximately 21 genus-level taxa. This was followed by the GW region, which harbored 17 unique genus-level species, while the JB region recorded the lowest diversity, with only two genus-level species identified (Fig. 6A). Among the commonly shared ones are *Lachnospira*, found both in GW and SD. In addition, *Wolbachia*, one of the popular taxa, was found in the CC region alongside other taxa. Further comparison was performed between the different regions to identify specific regional biomarkers. The 16S rRNA method can be used to determine the composition of microorganisms that make up less than 10% of the total microbiota. When comparing the microbiome of each region, the microbiome found to be region-specific was mostly found at a proportion of 10% or less (based on abundance). For example, seven taxa were commonly found between the SD, GB, GW, and CC regions, as well as between the CC, JN, JB, and JJ regions. Similarly, between GW, JN, JB, and SD regions with 5.3%, 8.1%, and 6.9%, respectively (Fig. S4).

### Microbial co-occurrence network

Co-occurrence networks enable us to deeply investigate the microbial relationship between the identified taxa from all the sampling locations. From our definitive finding, a significant correlation was found in the JN1, JB, and GN2 regions. Further significant correlation was observed between the GG and JN2 regions. Finally, despite the diverse results found from the JJ data, a significant correlation was found between the JJ and SD2 regions. This is not surprising considering that both locations are labeled as islands (Fig. 6B).

### Relationship between mosquito collection environment and microbiome

**Fig 7:**
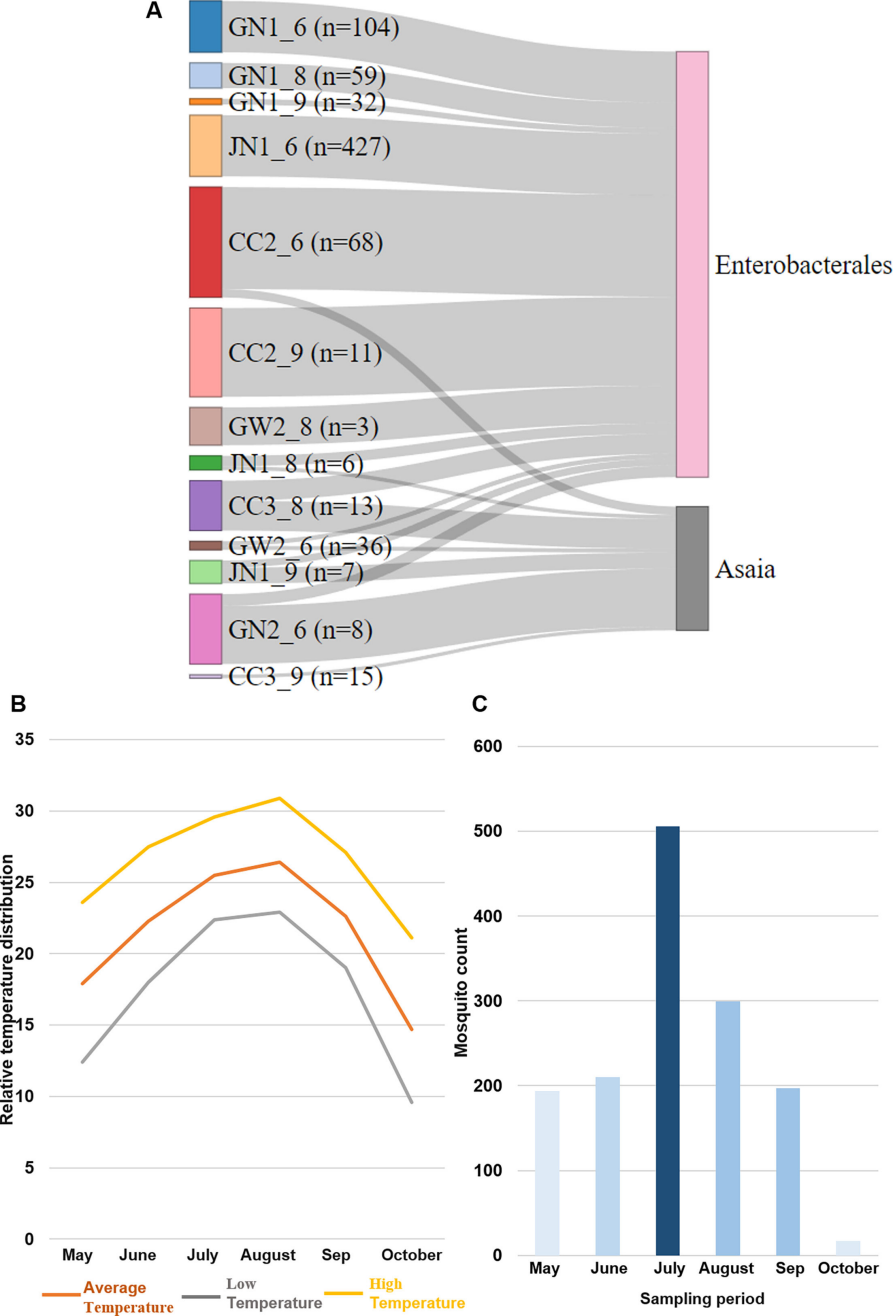
(**A**) Sankey diagram was generated using the NetworkD3 R-package, showing an illustration of the correlation between mosquito collection and certain microbiome (*Asaia*, *Enterobacterales*). The “n” stands for the number of mosquito samples collected. (**B**) Temperature variation occurs within months from May to October. Higher temperatures and precipitation were observed in July and August. (**C**) This indicates the mosquito count during the collection months. There is an increase in the number of mosquitoes during July and August.

A significant correlation was found between the number of mosquitoes caught and the specific microbiome. In areas where mosquito collection was reduced, there was a tendency for *Enterobacterales* to decrease and for *Asaia* to increase. However, additional research is needed as other microbiome ratios and environments may also affect mosquito population density (Fig. 7A). *Asaia* bacteria are described as potential symbionts of several mosquito species, with a specific positive effect on improving host performance mediated by interactions with other bacteria (25). Furthermore, importantly, *Asaia* can speed up the development rate, hypothetically by modulating host transcription genes (30). *Enterobacterales* bacteria, particularly *Enterobacter cloacae* and *Serratia marcescens*, are commonly found in mosquito midguts and have been shown to reduce Plasmodium development. These symbionts offer promising potential as paratransgenic agents for blocking pathogenic transmission (31). These, in addition, are being found among the six commonly found taxa between Korea and Canada (Fig. S5), making us more focused on correlating the two taxa with another environmental factor, for pathogenic and mosquito development potential.

In addition, considering mosquitoes are ectothermic animals, their environment directly influences them. Consequently, their microbiome can change depending on environmental factors. From our definitive finding, the months of June and September showed similar microbiome distribution patterns but were quite different in August. In June and September, both temperature and precipitation were almost similar, while an increase in temperature and precipitation was observed in July and August (Fig. 7B and C). Larger variations may change the microbial environment of the mosquito’s aquatic habitat. Thus, spatiotemporal investigation of the microbiome is essential for marker development.

For the GG region, the collection volume did not change with the collection date, but the microbiome composition changed significantly. In June, the microbiome was composed mainly of *Spiroplasma* and *Dickeya*, while a more diverse microbiome was observed in August, including *Dickeya*, *Enterobacterales*, *Asaia*, and *Pseudomonas*. In SD1, the highest mosquito collection occurred in August, which was negatively correlated with the proportion of *Spiroplasma*. Additionally, compared to June and September, the proportion of *Asaia* was higher in August. In addition, the dominant microbiome in June and September was *Spiroplasma*, but a more diverse microbiome was identified in September. SD2 was a region where the clustering changed monthly, and the microbiome proportions varied each month. *Spiroplasma*, *Dickeya*, and *Enterobacterales* were the dominant genera in June, August, and September, respectively.

For CC2 and 3 regions, the collection times did not greatly affect their microbiome composition, unlike in CC1, where the collection volume dropped significantly in September. CC1 generally had a higher proportion of *Dickeya*, but this changed with the collection time, and in September, the proportion of *Asaia* was recorded as the highest. In addition, in CC2, *Enterobacterales* dominated in June and September but were completely absent in August. Similar to SD, GB1 shows the highest proportion of *Spiroplasma* in June and is absent in August. Their composition and proportions resembled those of the September samples. Largely, the August and September samples in GB1 showed a balanced microbiome with *Dickeya*, *Enterobacterales*, *Asaia*, *Pseudomonas*, and *Acinetobacter*. The mosquito collection in GN1 gradually decreased over time. GN1 showed a similar microbiome composition to GB, with the difference in the presence of Pseudomonas only in August. On the other hand, the proportion of *Enterobacterales* gradually decreased. However, in JN1, there was a significant difference between June and the August-September period. For example, in June, the proportion of *Enterobacterales* was high, while *Asaia* and *Acinetobacter* were absent. On the other hand, in August and September, the proportion of *Acinetobacter* was higher than that of *Enterobacterales*, and *Asaia* was also found. The proportion of *Spiroplasma* was also lower in August and September compared to June. Contrary to JN1, in JN2, the proportion of *Dickeya* was similar in June and September, but *Enterobacterales* and *Spiroplasma* were dominant in each respective month.

Lastly, the JJ mosquito collection occurred only in September. The mosquito population decreased in September with an increase in *Asaia* and a decrease in *Enterobacterales. Asaia* is known to produce antimalarial proteins in Anopheles, which strengthen the immune system, and it is also correlated with *Wolbachia*. Therefore, further research on the relationship between *Asaia* and mosquitoes is expected to contribute to advancements in vector-borne disease control.

### Comparison of the mosquito microbiome between Korea and Canada

Following a similar pattern observed in studies comparing regional microbiome distributions in mosquitoes across Canada and other countries. We compared our findings with microbiome profiles from overseas mosquito populations using publicly available data sets. This comparative analysis revealed both shared and unique microbial taxa between native and foreign mosquito communities. Specifically, *Dickeya*, *Lactococcus*, and members of the Acetobacteraceae family were uniquely identified in native mosquito samples collected from the Republic of Korea (Fig. S5). Among these, *Dickeya* was consistently present across multiple native collection sites and absent from foreign data sets, highlighting its potential as a biomarker for region-specific mosquito microbiota.

Conversely, six taxa were found to be consistently present in both native and overseas mosquito populations: *Spiroplasma, Enterobacterales, Asaia, Pseudomonas, Acinetobacter*, and *Microbacterium*. *Spiroplasma* appeared in greater abundance in native samples, likely due to localized environmental and host factors. Among these consistently present taxa, *Enterobacterales* and *Asaia* were used for comparing the relationship between mosquito collection and microbiome. From our definitive findings, we realized that, in areas where mosquito collection was reduced, there was a tendency for *Enterobacterales* to decrease and for *Asaia* to increase (Fig. 7A).

**Fig 8:**
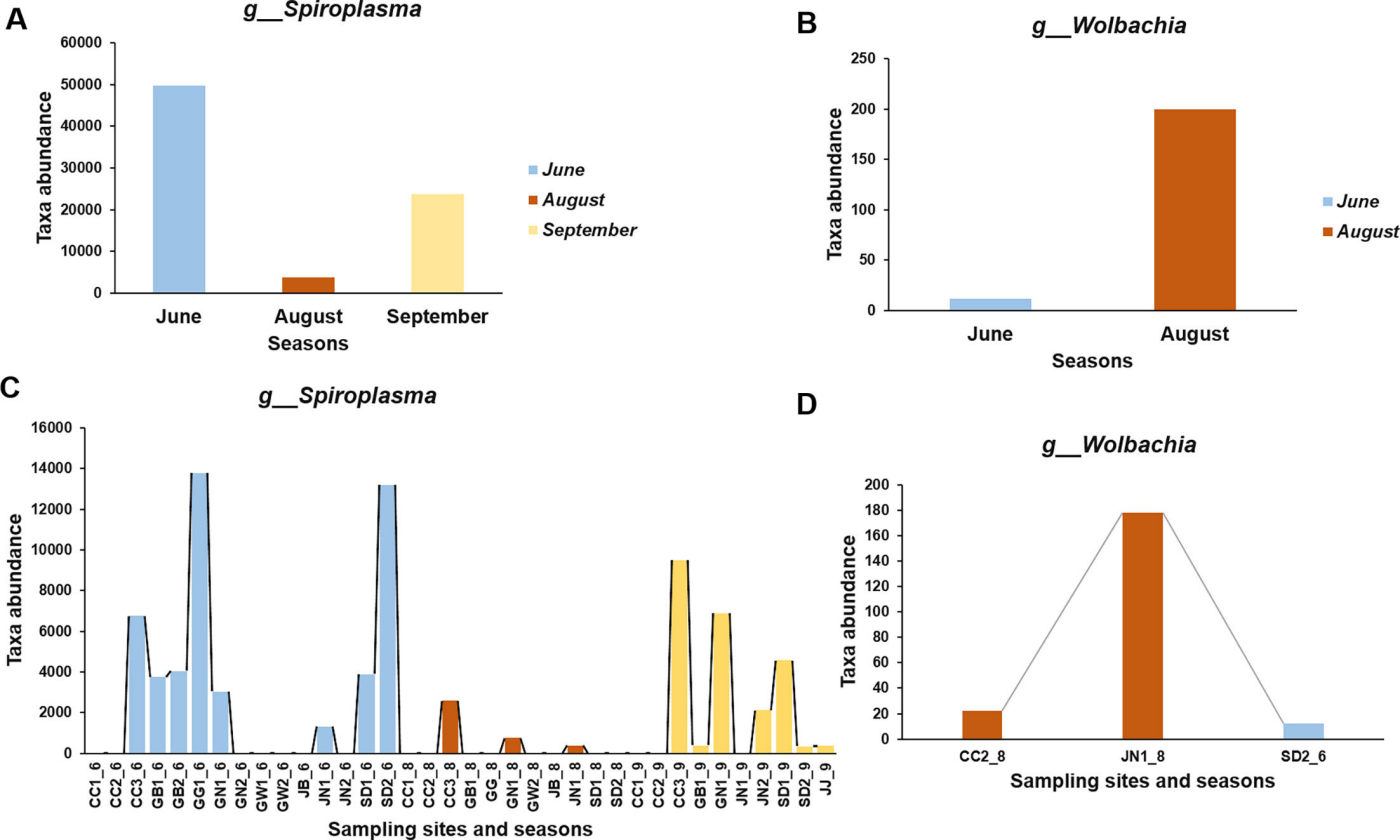
(**A**) Relative abundance of *Spiroplasma* across three sampling periods (June, August, and September). *Spiroplasma* showed the highest abundance in June, followed by a decline in August and partial resurgence in September. (**B**) Relative abundance of *Wolbachia* across sampling periods. *Wolbachia* was only detected in June and August. The limited distribution of *Wolbachia* suggests its restricted prevalence among regional populations of *Aedes vexans,* potentially reflecting environmental or host-specific influences. (**C**) The *Spiroplasma* genus distribution was found in only 15 sampling sites among the 16 sites mentioned in June, 11 in August, and 10 in September. The most affected regions include Gyeonggi-do (GW), Sudo (SD), and Chungcheong (CC). (**D**) The *Wolbachia* genus distribution was found in only three sampling sites among the 16 sites mentioned. The affected regions include the following: Chungcheong (CC) in August, Jeju (JJ) in August, and Sudo (SD) in June.

*Pseudomonas* was notably more abundant in overseas mosquitoes. These differences may reflect distinct ecological pressures, dietary influences, and host microbiota coevolution in different geographical contexts. In contrast, several taxa were exclusive to the overseas mosquito microbiome with very low abundance rates or were completely absent, including *Ralstonia*, *Bacteroidetes, Rickettsiella, Bradyrhizobium, Wolbachia*, and *Gluconobacter*. Among these, *Wolbachia*, a well-documented endosymbiont with vector control potential (32), was found in only three sampling sites in native *Aedes vexans*, indicating a possible lack of natural infection in this species within the Korean ecosystem (Fig. 8B).

Taken together, these results underscore the unique microbial signature of native mosquitoes and suggest that *Dickeya* may serve as a useful molecular marker for distinguishing local from non-native mosquito populations. However, it remains to be determined whether *Dickeya* establishes a stable, vertically transmitted symbiosis or is transiently acquired from the environment. Further longitudinal and functional studies are warranted to investigate the ecological role and persistence of *Dickeya* within native mosquito microbiomes.

On the other hand, we further identify the abundance and distribution of *Spiroplasma* and *Wolbachia* taxa using Korean samples, considering their pathogen-specific potential. From our definitive finding, *Wolbachia* was found in only three locations, including CC2, JN1 in August, and the SD2 site in June. Its absence in September and low overall occurrence suggest that *Wolbachia* may not be stably maintained in these mosquito populations.

Unlike *Wolbachia, Spiroplasma* shows a wider distribution throughout the three seasons, with the highest abundance recorded in June, a drastic decline in August, and a moderate resurgence in September. Spatially, *Spiroplasma* was found mostly dominant in samples from GG1_6, SD2_6, and CC3_6, indicating possible environmental or host-related influences on its prevalence (Fig. 8A through D).

## DISCUSSION

Mosquitoes, particularly blood-feeding species such as *Aedes vexans*, host a wide array of microbial communities. However, the specific biotic and abiotic factors contributing to microbial dysbiosis in these vectors remain poorly understood. This study aimed to investigate how environmental parameters, specifically sampling location, season, temperature, and humidity, shape the microbiome composition of *Aedes vexans* in South Korea, with implications for vector competence and arboviral disease risk.

Expanding on previous research limited to 12 northern sites (24), this study included 16 diverse regions across South Korea, including an island environment, to capture broader ecological variations. The analysis revealed that the mosquito microbiota was predominantly composed of Proteobacteria and Firmicutes, consistent with earlier studies (33–35). This taxonomic pattern suggests certain ecological traits of Proteobacteria may support their persistence in mosquitoes across different habitats (36).

Host sampling location emerged as a significant determinant of microbiome composition, likely reflecting local climate conditions, availability of blood and nectar sources, and larval habitat characteristics. These environmental influences have been widely supported by earlier work (37, 38). As such, most of these bacterial taxa could be acquired from vertebrate blood-meal hosts, larval environment, among others (35). Our study found that many dominant taxa, such as *Bacillus, Pseudomonas,* and *Acinetobacter,* are generalists present in both aquatic and terrestrial environments (39–41). In addition, *Acinetobacter* is commonly found in nectar with a relative abundance ranging between 49% and 90% in certain plants (39). It is worth knowing that the *Corynebacterium,* known to be associated with the skin of humans and animals, was absent or less abundant in our mosquito samples (40). This might be related to the sampling sites, as they are closer to the sea sites. Similar to observation in *Drosophila* species, varying environmental conditions can host distinct bacterial communities, leading mosquitoes from different ecological niches to acquire diverse microbiota (42).

Across all regions, eight major genera dominated the mosquito microbiota: *Dickeya, Spiroplasma, Enterobacterales, Asaia, Pseudomonas, Acinetobacter, Enterococcus,* and *Lactococcus*. Notably, *Wolbachia,* a genus known for its antiviral properties, was absent in our samples, despite being reported as dominant in *Aedes albopictus* in other studies (43–45). Given its known role in suppressing arboviral replication, the absence of *Wolbachia* might signal increased vector susceptibility to viruses such as WNV in these populations (34, 46). In addition to the biological importance of *Wolbachia,* which is capable of inhibiting arbovirus infection of *Aedes* spp. (44). Normally, the microbiome has direct action against arboviruses, mainly due to the ability of *Wolbachia* species to block the viral genome replication, added to the competition for resources within the mosquito organism (45). In addition, *Wolbachia* has been used to control the transmission of pathogens through artificial infection, which renders many mosquitoes refractory to infection and transmission of diverse pathogens (47). This phenomenon was not feasible in certain mosquito species, such as *Culex tarsalis* (47). Although *Culex tarsalis* is highly competent for WNV and western equine encephalitis virus (48, 49), experimental removal of *Wolbachia* in *Culex* spp. suggests that the endosymbiont may disrupt WNV dynamics in mosquito tissues (50, 51). In a separate study involving *Aedes albopictus*, Tsai et al. reported that native *Wolbachia* was associated with reduced dengue virus in mosquito tissues. These findings suggest that the absence of *Wolbachia* could potentially facilitate the increased replication of related viruses, such as WNV (52). *Wolbachia’s* role is not only restricted to reducing mosquitoes’ ability to transmit viruses but also lowering mosquito populations (53). Overall, *Wolbachia* and other microbiota in mosquitoes varied across seasons in WNV mosquito vectors, and their infections were found to be negatively correlated with WNV in these mosquitoes (36).

For *Enterobacterales* abundance, blood meals stimulate its proliferation during the *Aedes aegypti* mosquito digestion, inducing a functional shift and suggesting a role in assisting blood metabolism (54). This highlights the possibility of blood-feeding activities from higher animals since some of our sampling was carried out near the cattle-rearing area.

The microbiome of JJ Island mosquitoes was uniquely dominated by Muribaculaceae, Lachnospiraceae*,* Bacteroides, and Muribaculum, taxa typically associated with mammalian hosts and previously linked to viral infections such as chikungunya (55). Their distinct composition likely reflects the region’s unique climate and land use and further supports the role of environmental context in shaping microbial communities (56). This was further validated through the microbial co-occurrence network analysis, which highlights a correlation between the microbiome of island-like (SD2) regions sharing similar environmental dynamics.

For context, we compared the Korean *Aedes vexans* microbiome with publicly available data sets from Canadian populations of the same species (36). Although WNV transmission risk is low in Canada, this contrast underscores the influence of local ecology and climate on microbial composition. The presence of *Dickeya, Lactococcus,* and *Acetobacteraceae* as unique to Korean mosquitoes suggests region-specific microbial signatures, which could serve as useful ecological markers. However, direct comparisons with *Aedes vexans* from WNV-endemic regions would be necessary to confirm these associations.

Studies have shown that mosquito microbiome diversity shifts along large-scale moisture gradients in the landscape (56). Furthermore, a reduction in bacterial diversity among *Culex* mosquitoes in urbanized areas with higher elevation was observed (57). Urbanization and developed land cover often lead to habitat alterations, including but not limited to changes in the availability and quality of mosquito breeding sites. Therefore, these artificial breeding sites can be influenced by various urbanization-related factors, including pollutants (58), as well as the urban heat island effect (59). Consequently, and as a result of these factors, they may exhibit distinct microbial communities when compared to their counterparts in the natural habitats.

In addition to habitat-based differences, seasonal shifts in microbiome composition were also evident. In June, *Spiroplasma* predominated, followed by *Dickeya* and *Enterobacterales*. As an endosymbiont, *Spiroplasma* has been linked to resistance against various parasites but may also cause virulence in male hosts under certain infections (60). In contrast, August samples exhibited a surge in *Dickeya, Dietzia, Tanticharoenia,* and *Acetobacteraceae* genera, largely absent in other months. This microbial shift coincided with the known seasonal peak of WNV in vectors like *Culex pipiens* as presented under Canadian environmental influence (36). Notably, the rise in temperature and precipitation during August is likely to alter the microbial environment of *Aedes vexans,* potentially leading to a drastic increase in the relative abundance of *Dickeya* by more than twofold. In contrast, a decline in the number of mosquitoes collected in September observed reflects natural seasonal fluctuations in mosquito density driven by environmental factors such as rainfall and temperature, among others. This is in line with similar seasonal reductions reported in previous vector ecology studies, whereby Benin recorded substantially higher mosquito abundance during the wet season than the dry season, reflecting strong seasonal variation in vector density (61, 62). Zhou et al. reported that the *Dickeya* genus comprises numerous pathogenic species that cause diseases in various crops (63). This highlights that the sampled mosquitoes might have direct contact with infected crops as their main source of nectar. While seasonal variation in microbiome composition may be influenced by local environmental factors such as temperature, humidity, and precipitation, our study did not include parallel meteorological data to test these relationships directly. Future studies integrating local climate data with the identified microbiome will be essential to confirm these associations.

Accumulating evidence shows that environmental habitats and climates seem to shape the abundance of *Dickeya* species in nature (63, 64). *Dickeya* has a pathogenic potential, believing that if *Dickeya* species are active within the mosquito gut, they could contribute to microbial dysbiosis, potentially affecting mosquito health and fitness, in addition to its vector competence potential (63). Alterations in the gut microbiome can influence a mosquito’s ability to transmit pathogens. The presence of *Dickeya* might modulate this competence, either enhancing or suppressing the transmission of diseases like malaria or dengue, among others (65). In a nutshell, the acquisition of *Dickeya* by mosquitoes could reflect environmental exposures, especially in areas where these bacteria are prevalent due to agricultural activities (66). For the sampling season, the genus *Dickeya,* traditionally known as a plant pathogen, was particularly abundant, especially during August, a period characterized by high temperature and precipitation (67, 68). This seasonal dominance, along with *Dickeya’*s known virulence and its potential to alter host microbial balance, suggests that it could act as a modulator of vector competence. Its high abundance may reflect nectar feeding on infected crops, reinforcing the link between the agricultural environment and mosquito microbiome composition (69). Given its absence in overseas populations, *Dickeya* may also serve as a potential biomarker for identifying native mosquito populations.

*Dietzia* is a promising paratransgenesis candidate, previously isolated from *Aedes albopictus* and shown to promote larval development (70). Paratransgenesis is a strategy for controlling vector-borne diseases that involves genetically modifying a host insect’s symbionts, such as bacteria, to express anti-pathogen molecules. This helps in reducing the insect’s ability to transmit a pathogen without directly harming the insect itself. Therefore, its ability to survive across a wide range of environmental conditions, along with its occasional association with human infections, highlights its dual potential as a vector tool and an emerging pathogen. A higher abundance of the family *Dietziaceae* was observed in the human-blood-fed mosquitoes compared to that of non-human-blood-fed mosquitoes (71).

Collectively, these findings demonstrate that mosquito microbiota are not static but are shaped by a complex interplay of seasonal, ecological, and geographic factors. Understanding these dynamics is crucial for identifying microbiome-based markers of vector populations and for informing microbiota-targeted vector control strategies in the context of WNV and other arboviral diseases.

### Conclusion

This study provides comprehensive insights into the spatial and temporal dynamics of the *Aedes vexans* microbiome across 16 regions in South Korea, revealing an interplay between environmental factors and microbial community structure. Seasonal and spatial differences in mosquito microbiome composition were evident in our study. These patterns suggest that environmental factors may influence bacterial community structure, although our analysis did not directly test these associations. Future work that integrates microbiome data with local climatic and ecological variables will be essential to clarify these potential interactions, especially in the Republic of Korea. Proteobacteria and Firmicutes, with notable seasonal and regional shifts in genera such as Dickeya, Spiroplasma, and Enterobacterales, dominated the mosquito microbiota. The prevalence of *Dickeya* during warm, wet periods (August) suggests environmental acquisition and potential as an ecological marker for interaction between mosquitoes and agricultural products. Comparative analyses showed that *Dickeya, Lactococcus*, and *Acetobacteraceae* were unique to Korean populations, distinguishing them from Canadian *Aedes vexans*. The absence of *Wolbachia*, a symbiont known to reduce arbovirus transmission, may increase local susceptibility to WNV. These findings highlight how geography, seasonality, and environment shape mosquito-associated microbiota and underscore their potential in vector surveillance, ecological monitoring, and microbiome-informed control strategies against emerging arboviruses such as WNV.

## Data Availability

The raw sequence and metadata generated were submitted to the NCBI BioProject database under accession number PRJNA1274072.

## SUPPLEMENTAL MATERIAL

Supplemental figures spectrum.02587-25-s0001.docx Fig. S1 to S5; Table S10.

Supplemental tables spectrum.02587-25-s0002.docx Tables S1 to S4.

Table S5 spectrum.02587-25-s0003.docx Concentration of the extracted DNA from each sampling site.

Supplemental material spectrum.02587-25-s0004.docx Tables S6 to S8.

Table S9 spectrum.02587-25-s0005.docx Number of valid reads obtained from each sample.

ASM does not own the copyrights to Supplemental Material that may be linked to, or accessed through, an article. The authors have granted ASM a non-exclusive, world-wide license to publish the Supplemental Material files. Please contact the corresponding author directly for reuse.
