# Supplementary material for: Spatiotemporal variation in the microbiome of Aedes vexans from Korea reveals regional markers linked to environmental risk factors
Source: Microbiol Spectr. 2026 Mar 31;14(5):e02587-25. doi: 10.1128/spectrum.02587-25 (PMC13141922; doi:10.1128/spectrum.02587-25)
Supplement: Supplemental figures — Fig. S1 to S5; Table S10. [file spectrum.02587-25-s0001.docx]

**Supplementary Figures**


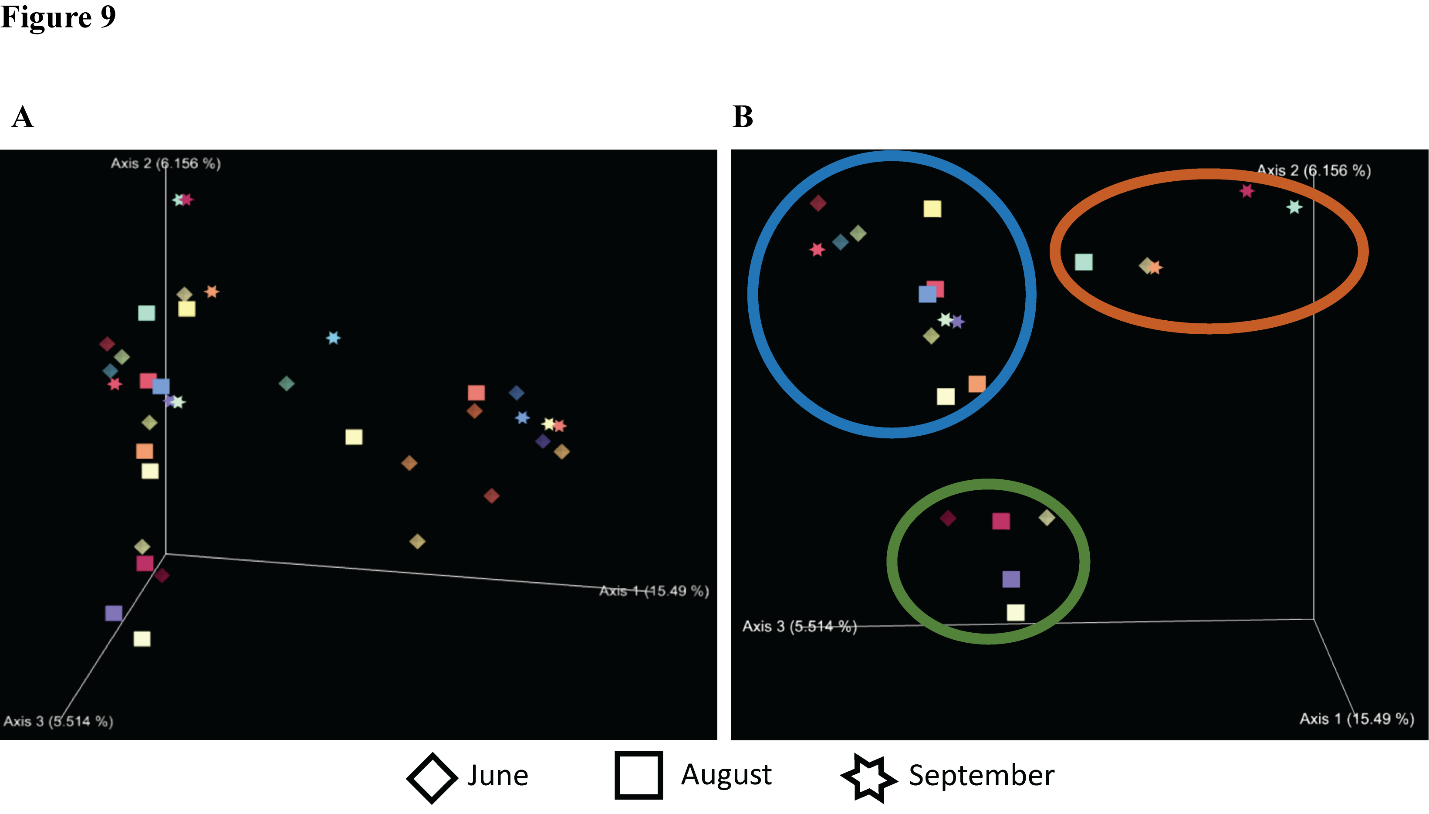


**Supplementary Fig. 1: (A)** Score plot for principal coordinate analysis (PCoA) of the bacterial community compositions at the genus level in the *Aedes vexans* gut using multivariate analysis method. The individual samples from each month had different shapes and were color coordinated to the respective region. A color gradient used ranges from the Brown start, for the CC1 region, to the Purple cube, for SD2. **(B)** Different regions were found sharing similar taxa and grouped into three coordinates based on the taxa's similarity. The result shows no effect of the sample timing on the taxa similarity based on the three groups.

**
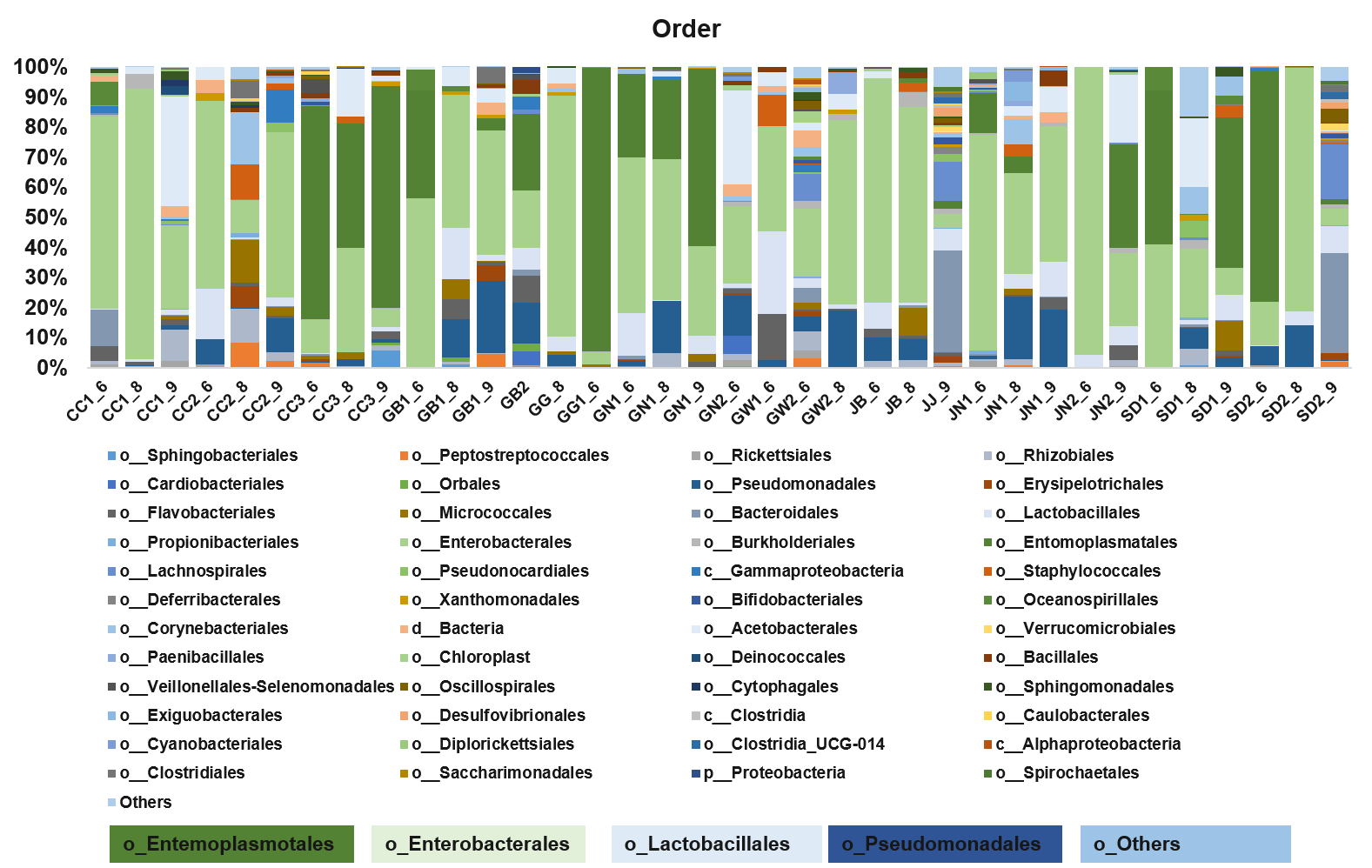
**

**
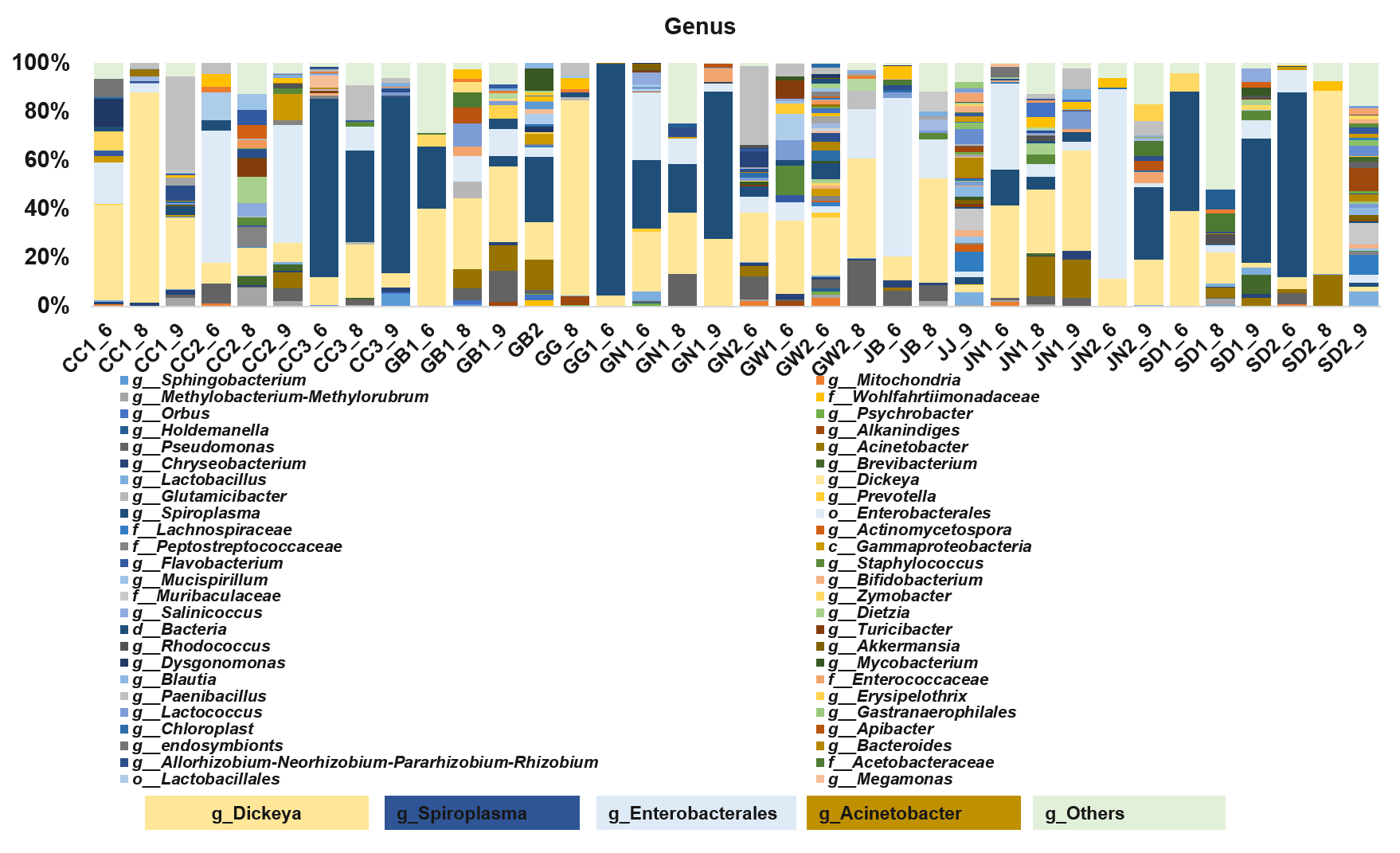
**

**Supplementary Fig. 2:** Stacked bar plots depict the distribution of microbiome composition by sampling sites at the order, and genus level. At the phylum level. Proteobacteria, Firmicutes, and Bacteroidota are the dominant taxa. In August, in addition to Actinobacteriota, Proteobacteria, Firmicutes, and Bacteroidota are the dominant taxa. While in September, Proteobacteria, Firmicutes, and Bacteroidota are the dominant taxa, which is similar to the June samples. JJ samples show a unique microbial distribution. The main regions include: Gangwon (GW); Gyeonggi (GG); Sudogwon (SW); Chubngcheong (CC); Gyeongbuk (GB); Jeonbuk (JB); Gyeongnam (GN); Jeonnam (JN); and Jeju (JJ). Jeju samples show an entirely different pattern of bacterial composition as compared with the other regions. Microbiome accounting for less than 1% were considered less prevalent and such summed up to the category of <1%.


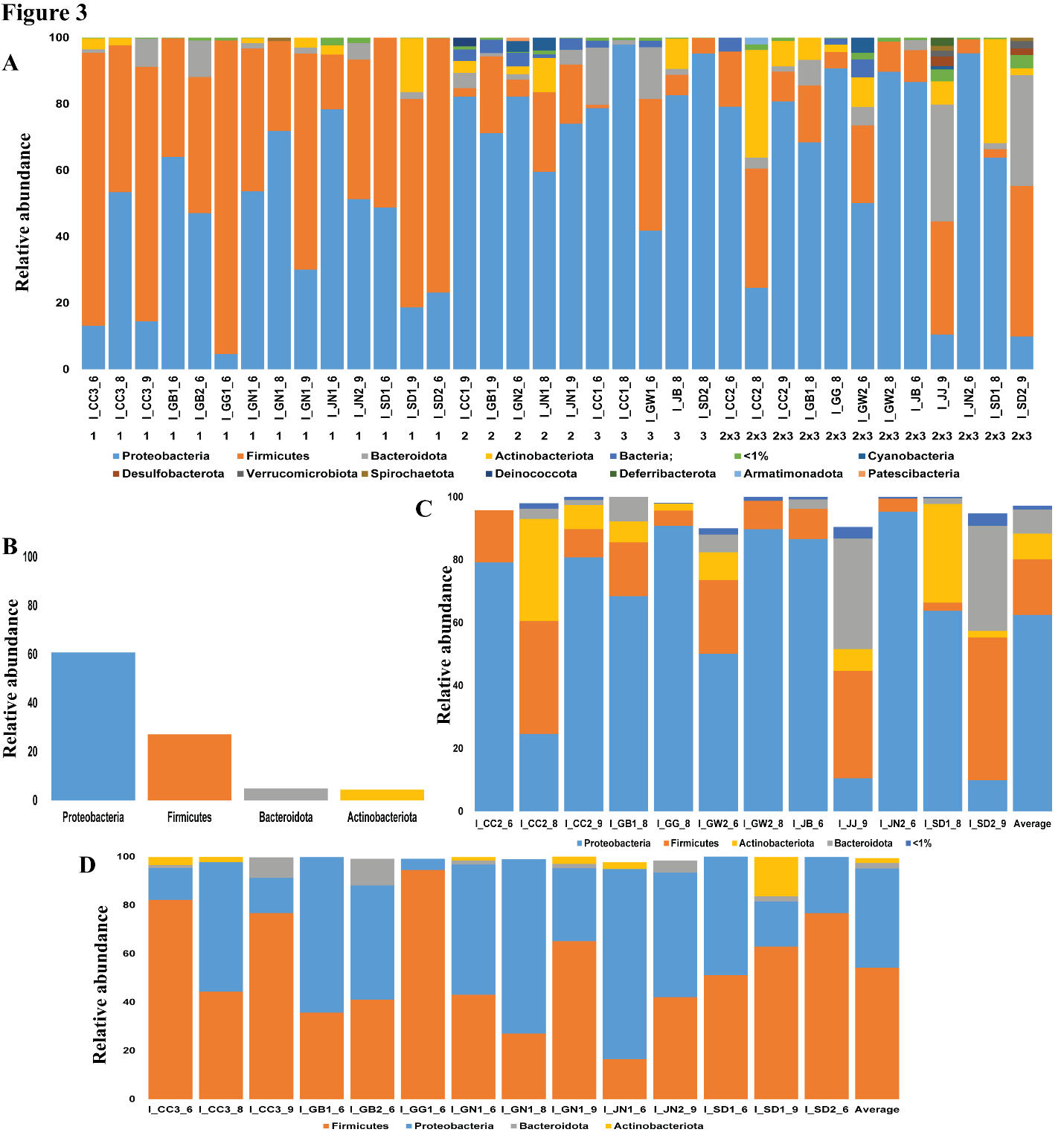


**Supplementary Fig. 3: (A)** Microbiome profiles of adult *Aedes vexans* mosquitoes at the phylum level. The microbiome distributions were made based on two different axes, including Axis-2 and Axis-3. **(B)** Microbiome profiles of adult *Aedes vexans* mosquitoes at the phylum level. The most abundant phylum were shown, indicating Proteobacteria with the highest abundance rate based on the average value. **(C)** Microbiome profiles of adult *Aedes vexans* mosquitoes at the phylum level. Samples in Axis-3 demonstrate different taxa distribution with a higher proportion of Actinobacteriota and Bacteroidota as compared to other samples. **(D)** Microbiome profiles of adult *Aedes vexans* mosquitoes at the phylum level. Samples in Axis-1 demonstrate different taxa distribution with a higher proportion of Firmicutes and Proteobacteria as compared to other samples. Added to that, samples with a higher proportion of Proteobacteria (GN1_8, JN1_6, JN2_9, etc.) are found located closer to Axis 2 and 3.


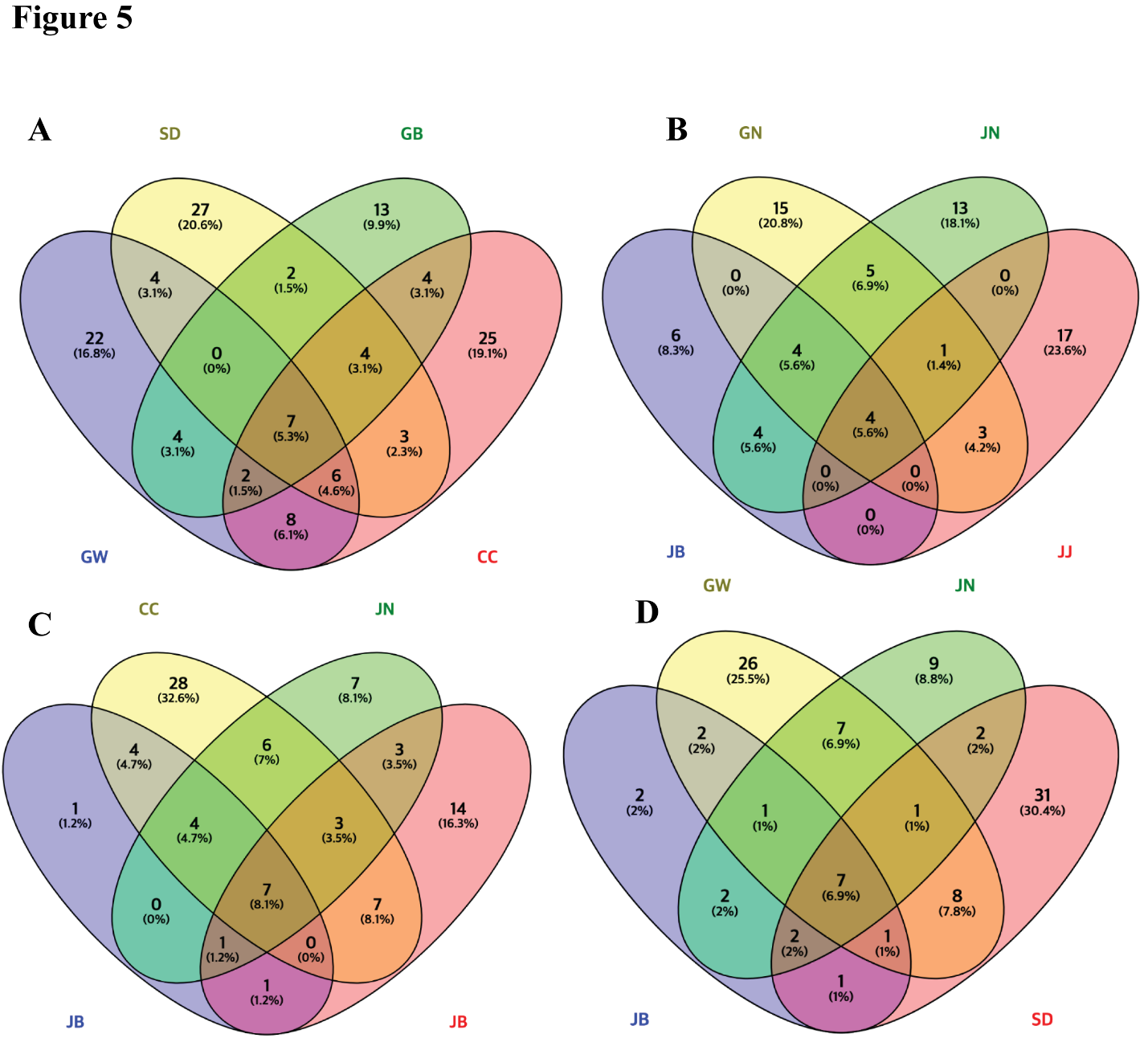


**Supplementary Fig. 4:** Comparison between the microbiome of different regions with one another. The commonly identified microbiome between each group is said to be the proposed region-specific microbiome. **(A)** SD, GB, GW, and CC compose 7 common bacteria occupying a total of 5.3% of the total microbiome. **(B)** GN, JN, JB, and JJ compose 4 common bacteria occupying a total of 5.6% of the total microbiome. **(C)** CC, JN, JB, and JJ compose 7 common bacteria occupying a total of 8.1% of the total microbiome. **(D)** GW, JN, JB, and SD compose 7 common bacteria occupying a total of 6.9% of the total microbiome. The microbiome found to be region-specific were mostly found at a proportion of less than 10%.


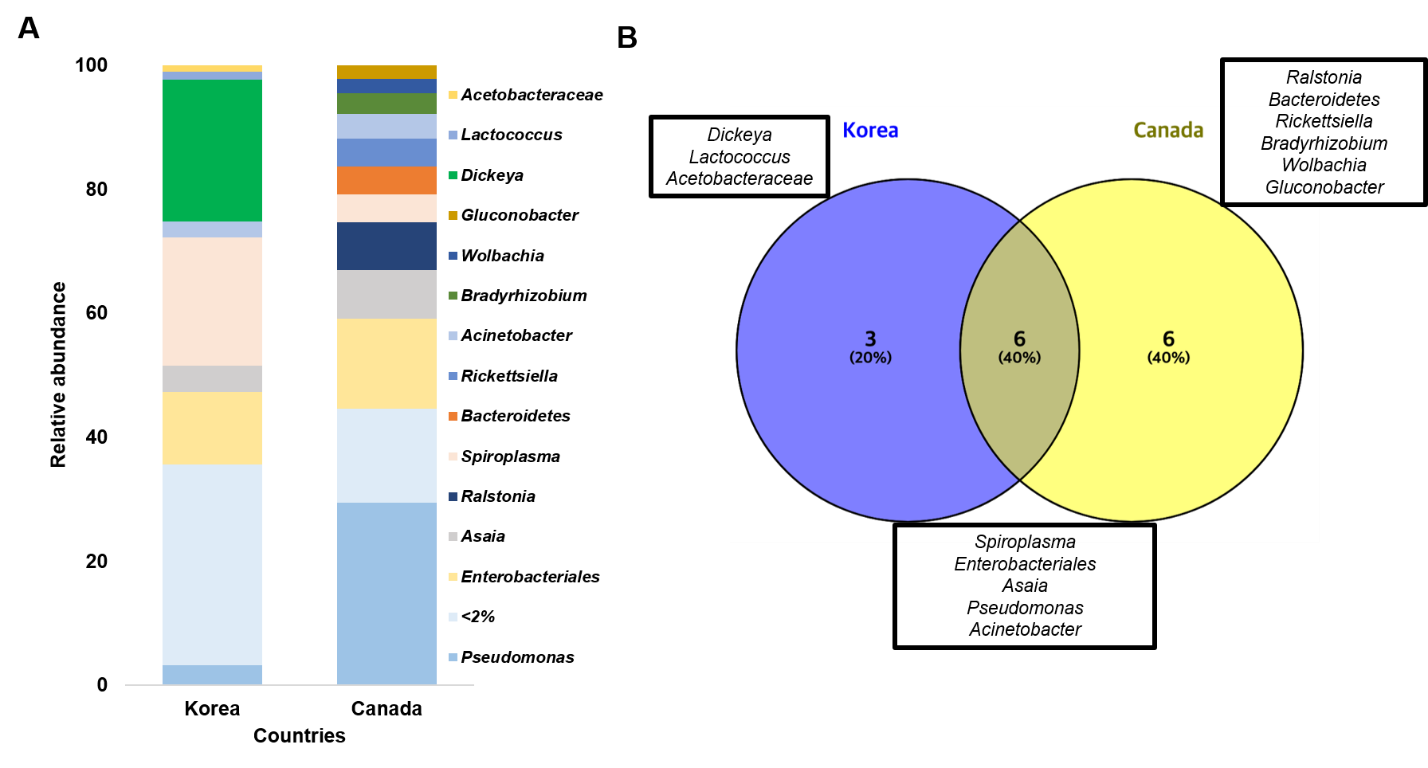


**Supplementary Fig. 5: (A)** Comparative analysis of microbiome profiles of adult *Aedes vexans* mosquitoes between South Korea (Korea) and Canada samples. **(B)** Venn diagram indicating both shared and unique taxa between South Korea (Korea) and Canada samples. Three and six unique taxa were found in Korea and Canada samples, respectively. On the other hand, six common taxa were found between the samples of Korea and Canada.

**Supplementary Table 10:** Region-specific microbial distributions at the genus-species level among various sampling sites.

| GW | SD | GG | CC | GB | JB | GN | JN |
| --- | --- | --- | --- | --- | --- | --- | --- |
| *Paenibacillus* | *Kineococcus* | *Alkanindiges* | *Salinicoccus* | *Erysipelothrix* | *Microbacterium* | *Ignatzschineria* | *Acetobacteraceae* |
| *Weissella* | *Streptomyces* | *Stenotrophomonas* | *Sphingobacterium* | *Proteus* | *Carnobacterium* | *Atopostipes* | *Cyanobacteriales* |
| *Empedobacter* | *Amycolatopsis* | *Corynebacterium* | *Pseudonocardia* | *Orbus* |  | *Curtobacterium* | *Rickettsiella* |
| *Chishuiella* | *Zymomonas* | *Glutamicibacter* | *Burkholderiales* | *Lysinibacillus* |  | *Massilia* | *Saccharibacillus* |
| *Providencia* | *Mycoavidus* |  | *Jeotgalicoccus* | *Acetobacter* |  | *Scytonema* | *Cutibacterium* |
| *Oxalobacteraceae* | *Williamsia* |  | *Deinococcus* | *Wohlfahrtiimonas* |  | *Spirochaetaceae* |  |
| *Fusicatenibacter* | *Lachnospiraceae* |  | *Armatimonas* | *Paraclostridium* |  | *Bacillales* |  |
| *Flavobacteriaceae* | *Oscillospiraceae* |  | *Wolbachia* | *Wohlfahrtiimonadaceae* |  |  |  |
| *Anaerostipes* | *Acetatifactor* |  | *Rhizobiaceae* | *Dysgonomonas* |  |  |  |
| *Alphaproteobacteria* | *Pseudokineococcus* |  | *Ornithobacterium* | *Terrisporobacter* |  |  |  |
| *Lachnospiraceae* | *Alistipes* |  | *Bacillaceae* | *Proteobacteria* |  |  |  |
| *1174-901-12* | *Dubosiella* |  | *Microlunatus* | *Kurthia* |  |  |  |
| *Faecalibacterium* | *Oscillibacter* |  | *Micrococcaceae* |  |  |  |  |
| *Peptostreptococcaceae* | *Streptococcus* |  | *Enhydrobacter* |  |  |  |  |
| *Butyricicoccus* |  |  | *Intrasporangiaceae* |  |  |  |  |
| *Holdemanella* |  |  | *Sphingomonadaceae* |  |  |  |  |
| *Oxyphotobacteria* |  |  | *Rhodobacteraceae* |  |  |  |  |
|  |  |  | *Lechevalieria* |  |  |  |  |
|  |  |  | *Spirosoma* |  |  |  |  |
|  |  |  | *Rummeliibacillus* |  |  |  |  |
|  |  |  | *Brevundimonas* |  |  |  |  |
