# Supplementary material for: Spatiotemporal variation in the microbiome of Aedes vexans from Korea reveals regional markers linked to environmental risk factors
Source: Microbiol Spectr. 2026 Mar 31;14(5):e02587-25. doi: 10.1128/spectrum.02587-25 (PMC13141922; doi:10.1128/spectrum.02587-25)
Supplement: Supplemental tables — Tables S1 to S4. [file spectrum.02587-25-s0002.docx]

***Supplementary information 1:*** Mosquito collection results during June. The largest number of mosquitoes were collected in Jeonnam 1, followed by Chungcheong and Gyeongnam provinces. The Jeonnam area has shown the lowest mosquito counts.

| **Sample name** | **English label** | **Region** | **Date** | **Mosquito count** | **Mosquito collection number** |
| --- | --- | --- | --- | --- | --- |
| Gangwon1 6 (June) | GW1_6 | GW1 | 6 | 10 | 20 |
| Gangwon2 6 (June) | GW2_6 | GW2 | 6 | 10 | 36 |
| Gyeong-gi 6 (June) | GG_6 | GG | 6 | 10 | 41 |
| Gyeongnam1 6 (June) | GN1_6 | GN1 | 6 | 10 | 104 |
| Gyeongnam2 6 (June) | GN2_6 | GN2 | 6 | 8 | 8 |
| Gyeongbug1 6 (June) | GB1_6 | GB1 | 6 | 10 | 16 |
| Gyeongbug2 6 (June) | GB2_6 | GB2 | 6 | 10 | 10 |
| Sudo1 6 (June) | SD1_6 | SD1 | 6 | 10 | 14 |
| Sudo2 6 (June) | SD2_6 | SD2 | 6 | 10 | 12 |
| Jeonnam1 6 (June) | JN1_6 | JN1 | 6 | 10 | 427 |
| Jeonnam2 6 (June) | JN2_6 | JN2 | 6 | 2 | 2 |
| Jeonbuk 6 (June) | JB_6 | JB | 6 | 10 | 23 |
| Chungcheong1 6 (June) | CC1_6 | CC1 | 6 | 10 | 218 |
| Chungcheong2 6 (June) | CC2_6 | CC2 | 6 | 10 | 68 |
| Chungcheong3 6 (June) | CC3_6 | CC3 | 6 | 10 | 89 |

***Supplementary information 2:*** Mosquito collection results during August. The largest number of mosquitoes were collected in Chungcheong, followed by Sudo 1 and Jeonbuk area. Chuncheong 2 area appear to have the least number of samples.

| **Sample name** | **English label** | **Region** | **Date** | **Mosquito count** | **Mosquito collection number** |
| --- | --- | --- | --- | --- | --- |
| Gangwon2 8(August) | GW2_8 | GW2 | 8 | 3 | 3 |
| Gyeong-gi 8(August) | GG_8 | GG | 8 | 10 | 35 |
| Gyeongnam1 8(August) | GN1_8 | GN1 | 8 | 10 | 59 |
| Gyeongbug1 8(August) | GB1_8 | GB1 | 8 | 7 | 7 |
| Sudo1 8(August) | SD1_8 | SD1 | 8 | 10 | 89 |
| Sudo2 8(August) | SD2_8 | SD2 | 8 | 2 | 2 |
| Jeonnam1 8(August) | JN1_8 | JN1 | 8 | 6 | 6 |
| Jeonbuk 8(August) | JB_8 | JB | 8 | 10 | 69 |
| Chungcheong1 8(August) | CC1_8 | CC1 | 8 | 10 | 315 |
| Chungcheong2 8(August) | CC2_8 | CC2 | 8 | 1 | 1 |
| Chungcheong3 8(August) | CC3_8 | CC3 | 8 | 10 | 13 |

***Supplementary information 3:*** Mosquito collection results during September. The largest number of mosquitoes were collected in Chungcheong 1. The overall number of mosquitoes collected was lower in September, while highest in August.

| **Sample name** | **English label** | **Region** | **Date** | **Mosquito count** | **Mosquito collection number** |
| --- | --- | --- | --- | --- | --- |
| Gyeongnam1 9(September) | GN1_9 | GN1 | 9 | 10 | 32 |
| Jeonbuk1 9(September) | GB1_9 | GB1 | 9 | 10 | 21 |
| Sudo1 9(September) | SD1_9 | SD1 | 9 | 9 | 9 |
| Sudo2 9(September) | SD2_9 | SD2 | 9 | 1 | 1 |
| Jeonnam1 9(September) | JN1_9 | JN1 | 9 | 7 | 7 |
| Jeonnam2 9(September) | JN2_9 | JN2 | 9 | 10 | 20 |
| Jeju 9(September) | JJ_9 | JJ | 9 | 3 | 3 |
| Chungcheong1 9(September) | CC1_9 | CC1 | 9 | 10 | 55 |
| Chungcheong2 9(September) | CC2_9 | CC2 | 9 | 10 | 11 |
| Chungcheong3 9(September) | CC3_9 | CC3 | 9 | 10 | 15 |

***Supplementary information 4:*** Average number of mosquitoes collected during the three sampling periods. The largest number of mosquitoes were collected in June, with the largest number being collected in Chungcheong 1 province.

| **Region** | **June** | **August** | **September** | **Average number of mosquitoes collected** |
| --- | --- | --- | --- | --- |
| Gangwon1 | 20 | X | X | 7 |
| Gangwon2 | 36 | 3 | X | 13 |
| Gyeong-gi | 41 | 35 | X | 25 |
| Gyeongnam1 | 104 | 59 | 32 | 65 |
| Gyeongnam2 | 8 | X | X | 3 |
| Gyeongbug1 | 16 | 7 | 21 | 15 |
| Gyeongbug2 | 10 | X | X | 3 |
| Sudo1 | 14 | 89 | 9 | 37 |
| Sudo2 | 12 | 2 | 1 | 5 |
| Jeonnam1 | 427 | 6 | 7 | 147 |
| Jeonnam2 | 2 | X | 20 | 7 |
| Jeonbuk | 23 | 69 | X | 31 |
| Jeju | X | X | 3 | 1 |
| Chungcheong1 | 218 | 315 | 55 | 196 |
| Chungcheong2 | 68 | 1 | 11 | 27 |
| Chungcheong3 | 89 | 13 | 15 | 39 |
