# Supplementary material for: Spatiotemporal variation in the microbiome of Aedes vexans from Korea reveals regional markers linked to environmental risk factors
Source: Microbiol Spectr. 2026 Mar 31;14(5):e02587-25. doi: 10.1128/spectrum.02587-25 (PMC13141922; doi:10.1128/spectrum.02587-25)
Supplement: Table S5 — Concentration of the extracted DNA from each sampling site. [file spectrum.02587-25-s0003.docx]

***Supplementary information 5A:*** Concentration of the extracted DNA from each sampling site.

| **No.** | **Sample ID** | **Conc.(ng/μl)** | **260/280** |
| --- | --- | --- | --- |
| 1 | GW2 | 34 | 2.01 |
| 2 | GG1 | 32.7 | 1.96 |
| 3 | GN1 | 40.4 | 1.94 |
| 4 | GN2 | 53.6 | 1.93 |
| 5 | GB1 | 59.1 | 1.92 |
| 6 | GB2 | 11.7 | 2.21 |
| 7 | SD1 | 63.8 | 1.9 |
| 8 | SD2 | 34.3 | 1.95 |
| 9 | JN1 | 24.8 | 1.98 |
| 10 | JN2 | 19.4 | 2.06 |
| 11 | JB1 | 19.1 | 2.07 |
| 12 | CC1 | 45.5 | 1.99 |
| 13 | CC2 | 97 | 1.9 |
| 14 | CC3 | 50.5 | 1.91 |
| 15 | GW1 | 38.1 | 1.88 |

***Supplementary information 5B:*** Concentration of the extracted DNA from each sampling site.

| **No.** | **Sample ID** | **Conc.(ng/μl)** | **260/280** |
| --- | --- | --- | --- |
| 1 | SD1_8 | 91.2 | 1.87 |
| 2 | SD2_8 | 43 | 1.91 |
| 3 | GG_8 | 71.1 | 1.88 |
| 4 | GW2_8 | 15.8 | 1.99 |
| 5 | CC1_8 | 22.8 | 1.95 |
| 6 | CC2_8 | 38.4 | 1.92 |
| 7 | CC3_8 | 146.7 | 1.86 |
| 8 | JB_8 | 135.4 | 1.85 |
| 9 | JN1_8 | 111.5 | 1.87 |
| 10 | GB1_8 | 103 | 1.87 |
| 11 | GN1_8 | 133.3 | 1.86 |

***Supplementary information 5C:*** Concentration of the extracted DNA from each sampling site.

| **No.** | **Sample ID** | **Conc.(ng/μl)** | **260/280** |
| --- | --- | --- | --- |
| 1 | SD1_9 | 43.9 | 1.89 |
| 2 | SD2_9 | 20.3 | 1.96 |
| 3 | CC1_9 | 23.4 | 1.94 |
| 4 | CC2_9 | 94.6 | 1.87 |
| 5 | CC3_9 | 63.7 | 1.88 |
| 6 | JN1_9 | 83.7 | 1.88 |
| 7 | JN2_9 | 141.7 | 1.86 |
| 8 | GB1_9 | 107.2 | 1.87 |
| 9 | JJ_9 | 66.7 | 1.88 |
| 10 | GN1_9 | 93.1 | 1.87 |

***Supplementary information 5D:*** Primer sequence information used for all the samples, amplifying V3-V4 regions.

|  |  |
| --- | --- |
| **Primer** | **Sequence (5-------->3)** |
| **16S v34_F** | TCGTCGGCAGCGTCAGATGTGTATAAGAGACAGCCTACGGGNGGCWGCAG |
|  | TCGTCGGCAGCGTCAGATGTGTATAAGAGACAGNCCTACGGGNGGCWGCAG |
|  | TCGTCGGCAGCGTCAGATGTGTATAAGAGACAGNNCCTACGGGNGGCWGCAG |
|  | TCGTCGGCAGCGTCAGATGTGTATAAGAGACAGNNNCCTACGGGNGGCWGCAG |
| **16S v34_R** | GTCTCGTGGGCTCGGAGATGTGTATAAGAGACAGGACTACHVGGGTATCTAATCC |
|  | GTCTCGTGGGCTCGGAGATGTGTATAAGAGACAGNGACTACHVGGGTATCTAATCC |
|  | GTCTCGTGGGCTCGGAGATGTGTATAAGAGACAGNNGACTACHVGGGTATCTAATCC |
|  | GTCTCGTGGGCTCGGAGATGTGTATAAGAGACAGNNNGACTACHVGGGTATCTAATCC |
