# Supplementary material for: Spatiotemporal variation in the microbiome of Aedes vexans from Korea reveals regional markers linked to environmental risk factors
Source: Microbiol Spectr. 2026 Mar 31;14(5):e02587-25. doi: 10.1128/spectrum.02587-25 (PMC13141922; doi:10.1128/spectrum.02587-25)
Supplement: Supplemental material — Tables S6 to S8. [file spectrum.02587-25-s0004.docx]

***Supplementary information 6:*** Number of reads obtained from each sample

| **Sample name** | **TotalReads** | **Q >= 30** | **(%)** | **Avg (Q >= 30)** |
| --- | --- | --- | --- | --- |
| CC1_6 | 63123 | 14371985 | 76.74% | 72.96% |
| CC1_6 | 63123 | 12945801 | 69.18% |  |
| CC2_6 | 171299 | 41578901 | 80.77% | 76.79% |
| CC2_6 | 171299 | 37424590 | 72.82% |  |
| CC3_6 | 90073 | 20988782 | 78.25% | 74.77% |
| CC3_6 | 90073 | 19088843 | 71.29% |  |
| GB1_6 | 185613 | 45204998 | 81.01% | 76.81% |
| GB1_6 | 185613 | 40433804 | 72.61% |  |
| GB2_6 | 154164 | 36995475 | 80.19% | 76.62% |
| GB2_6 | 154164 | 33667093 | 73.04% |  |
| GG1_6 | 131938 | 31635356 | 79.94% | 75.97% |
| GG1_6 | 131938 | 28436915 | 72.01% |  |
| GN1_6 | 165537 | 39845842 | 80.10% | 76.10% |
| GN1_6 | 165537 | 35807574 | 72.11% |  |
| GN2_6 | 101369 | 24039332 | 79.44% | 74.24% |
| GN2_6 | 101369 | 20859110 | 69.04% |  |
| GW2_6 | 159477 | 34916953 | 77.79% | 73.34% |
| GW2_6 | 159477 | 30923511 | 68.89% |  |
| JB1_6 | 170931 | 40651042 | 79.38% | 75.43% |
| JB1_6 | 170931 | 36553339 | 71.48% |  |
| JN1_6 | 118570 | 27539885 | 77.85% | 74.46% |
| JN1_6 | 118570 | 25106797 | 71.07% |  |
| JN2_6 | 160118 | 36303666 | 75.49% | 73.91% |
| JN2_6 | 160118 | 34733678 | 72.33% |  |
| SD1_6 | 154370 | 36894212 | 79.57% | 75.51% |
| SD1_6 | 154370 | 33079122 | 71.46% |  |
| SD2_6 | 162149 | 39058738 | 80.32% | 75.82% |
| SD2_6 | 162149 | 34618475 | 71.33% |  |

***Supplementary information 7:*** Number of reads obtained from each sample

| **Sample name** | **TotalReads** | **Q >= 30** | **(%)** | **Avg (Q >= 30)** |
| --- | --- | --- | --- | --- |
| CC1_8 | 121905 | 33473676 | 91.32% | 83.56% |
| CC1_8 | 121905 | 27745459 | 75.81% |  |
| CC2_8 | 69788 | 17617441 | 89.63% | 81.16% |
| CC2_8 | 69788 | 14311234 | 72.70% |  |
| CC3_8 | 79879 | 21819840 | 90.93% | 83.16% |
| CC3_8 | 79879 | 18056830 | 75.38% |  |
| GB1_8 | 93946 | 25466264 | 90.14% | 83.37% |
| GB1_8 | 93946 | 21605289 | 76.59% |  |
| GG_8 | 122167 | 33479796 | 91.11% | 83.67% |
| GG_8 | 122167 | 27959671 | 76.23% |  |
| GN1_8 | 68686 | 18665626 | 90.54% | 83.03% |
| GN1_8 | 68686 | 15549062 | 75.53% |  |
| GW2_8 | 62405 | 16947960 | 90.41% | 82.04% |
| GW2_8 | 62405 | 13792634 | 73.67% |  |
| JB_8 | 93487 | 25493262 | 90.71% | 82.62% |
| JB_8 | 93487 | 20906926 | 74.53% |  |
| JJ_8 | 75285 | 19441575 | 88.95% | 80.50% |
| JJ_8 | 75285 | 15767309 | 72.06% |  |
| JN1_8 | 90484 | 24585893 | 90.41% | 82.94% |
| JN1_8 | 90484 | 20494275 | 75.48% |  |
| SD1_8 | 58092 | 14192935 | 89.41% | 81.43% |
| SD1_8 | 58092 | 11687055 | 73.45% |  |
| SD2_8 | 95295 | 25994842 | 90.71% | 83.51% |
| SD2_8 | 95295 | 21827763 | 76.31% |  |

***Supplementary information 8:*** Number of reads obtained from each sample

| **File Name** | **TotalReads** | **Q >= 30** | **(%)** | **Avg (Q >= 30)** |
| --- | --- | --- | --- | --- |
| CC1_9 | 92631 | 25281334 | 90.85% | 83.39% |
| CC1_9 | 92631 | 21091886 | 75.94% |  |
| CC2_9 | 58741 | 15854065 | 90.30% | 82.52% |
| CC2_9 | 58741 | 13102921 | 74.73% |  |
| CC3_9 | 106576 | 29237832 | 91.27% | 83.65% |
| CC3_9 | 106576 | 24303650 | 76.03% |  |
| GB1_9 | 117421 | 31795645 | 90.10% | 82.77% |
| GB1_9 | 117421 | 26581903 | 75.44% |  |
| GN1_9 | 162412 | 44933494 | 91.94% | 84.29% |
| GN1_9 | 162412 | 37375580 | 76.64% |  |
| JN1_9 | 131232 | 35870956 | 90.91% | 83.42% |
| JN1_9 | 131232 | 29911564 | 75.94% |  |
| JN2_9 | 81841 | 20567168 | 90.06% | 82.50% |
| JN2_9 | 81841 | 17124636 | 74.94% |  |
| SD1_9 | 77193 | 21022183 | 90.61% | 82.38% |
| SD1_9 | 77193 | 17170683 | 74.14% |  |
| SD2_9 | 85318 | 22420402 | 89.58% | 81.72% |
| SD2_9 | 85318 | 18489664 | 73.85% |  |
