# Supplementary material for: Spatiotemporal variation in the microbiome of Aedes vexans from Korea reveals regional markers linked to environmental risk factors
Source: Microbiol Spectr. 2026 Mar 31;14(5):e02587-25. doi: 10.1128/spectrum.02587-25 (PMC13141922; doi:10.1128/spectrum.02587-25)
Supplement: Table S9 — Number of valid reads obtained from each sample. [file spectrum.02587-25-s0005.docx]

***Supplementary information 9:*** Number of valid reads obtained from each sample

| **Sample-id** | **Input** | **Filtered** | **Percentage of input passed filter** |
| --- | --- | --- | --- |
| I_CC1_6 | 146,687 | 75,733 | 52 |
| I_CC1_8 | 121,905 | 98,650 | 81 |
| I_CC1_9 | 92,631 | 73,844 | 80 |
| I_CC2_6 | 171,299 | 108,788 | 64 |
| I_CC2_8 | 69,788 | 42,780 | 61 |
| I_CC2_9 | 58,741 | 44,575 | 76 |
| I_CC3_6 | 163,244 | 83,619 | 51 |
| I_CC3_8 | 79,879 | 63,750 | 80 |
| I_CC3_9 | 106,576 | 86,476 | 81 |
| I_GB1_6 | 185,613 | 119,398 | 64 |
| I_GB1_8 | 93,946 | 75,904 | 81 |
| I_GB1_9 | 117,421 | 92,855 | 79 |
| I_GB2 | 154,164 | 95,068 | 62 |
| I_GG_8 | 122,167 | 99,390 | 81 |
| I_GG1_6 | 131,938 | 77,530 | 59 |
| I_GN1_6 | 165,537 | 100,757 | 61 |
| I_GN1_8 | 68,686 | 54,373 | 79 |
| I_GN1_9 | 162,412 | 134,097 | 83 |
| I_GN2_6 | 235,598 | 121,767 | 52 |
| I_GW1_6 | 89,257 | 78,564 | 88 |
| I_GW2_6 | 874,071 | 388,822 | 44 |
| I_GW2_8 | 62,405 | 48,023 | 77 |
| I_JB_6 | 170,931 | 99,974 | 58 |
| I_JB_8 | 93,487 | 72,637 | 78 |
| I_JJ_9 | 75,285 | 50,882 | 68 |
| I_JN1_6 | 251,959 | 135,384 | 54 |
| I_JN1_8 | 90,484 | 72,205 | 80 |
| I_JN1_9 | 131,232 | 106,164 | 81 |
| I_JN2_6 | 160,118 | 75,938 | 47 |
| I_JN2_9 | 81,841 | 50,787 | 62 |
| I_SD1_6 | 154,370 | 90,173 | 58 |
| I_SD1_8 | 58,092 | 32,768 | 56 |
| I_SD1_9 | 77,193 | 59,903 | 78 |
| I_SD2_6 | 162,149 | 96,524 | 60 |
| I_SD2_8 | 95,295 | 77,077 | 81 |
| I_SD2_9 | 85,318 | 61,847 | 72 |
